# Extraction of some pesticides from grape juice samples using an amide-based polymer as a sorbent followed by gas chromatographic determination

**DOI:** 10.1039/d6ra01736c

**Published:** 2026-10-26

**Authors:** Fatemeh Ershad, Mir Ali Farajzadeh, Sina Mohammad Mehri, Mohammad Reza Afshar Mogaddam

**Affiliations:** a Department of Analytical Chemistry, Faculty of Chemistry, University of Tabriz Tabriz Iran mafarajzadeh@yahoo.com mafarajzadeh@tabrizu.ac.ir +98 41 33340191 +98 41 33393084; b Engineering Faculty, Near East University North Cyprus, Mersin 10 99138 Nicosia Turkey; c Food and Drug Safety Research Center, Pharmaceutical Sciences Institute, Tabriz University of Medical Sciences Tabriz Iran; d New Material and Green Chemistry Research Center, Khazar University 41 Mehseti Street Baku AZ1096 Azerbaijan; e Pharmaceutical Analysis Research Center, Pharmaceutical Sciences Institute, Tabriz University of Medical Sciences Tabriz Iran

## Abstract

A sensitive and efficient analytical method based on dispersive micro solid phase extraction combined with dispersive liquid–liquid microextraction followed by gas chromatography–flame ionization detection was developed for the determination of haloxyfop-R-methyl, oxadiazon, diniconazole, tebuconazole, and difenoconazole in grape juice samples. An organic polymer was synthesized through a facile polyamidation reaction of benzene-1,3,5-tricarboxylic acid and ethylenediamine and employed as an efficient sorbent. The synthesized material was characterized by routine analyses. Key parameters affecting both extraction steps were systematically optimized. Under optimum conditions, the method showed limits of detection between 2.0 and 2.58 µg L^−1^, limits of quantification of 6.6–8.51 µg L^−1^, and wide linear ranges up to 6000 µg L^−1^ with excellent coefficients of determination (*r*^2^ = 0.9971–0.9990). High enrichment factors (365–495) and extraction recoveries (73–99%) were obtained with good precision (intra- and inter-day relative standard deviations ≤ 4.42%). Matrix effect evaluation in real grape juice samples revealed negligible interference, with relative recoveries in the range of 88–119%. In addition, the sorbent demonstrated acceptable reusability over four consecutive extraction cycles (RSD ≤ 15%). The proposed method provided a simple, cost-effective, and reliable approach for the determination of pesticide residues in complex beverage matrices.

## Abstract 2

A sensitive and efficient analytical method based on dispersive micro solid phase extraction combined with dispersive liquid–liquid microextraction followed by gas chromatography–flame ionization detection was developed.

## Graphical abstract

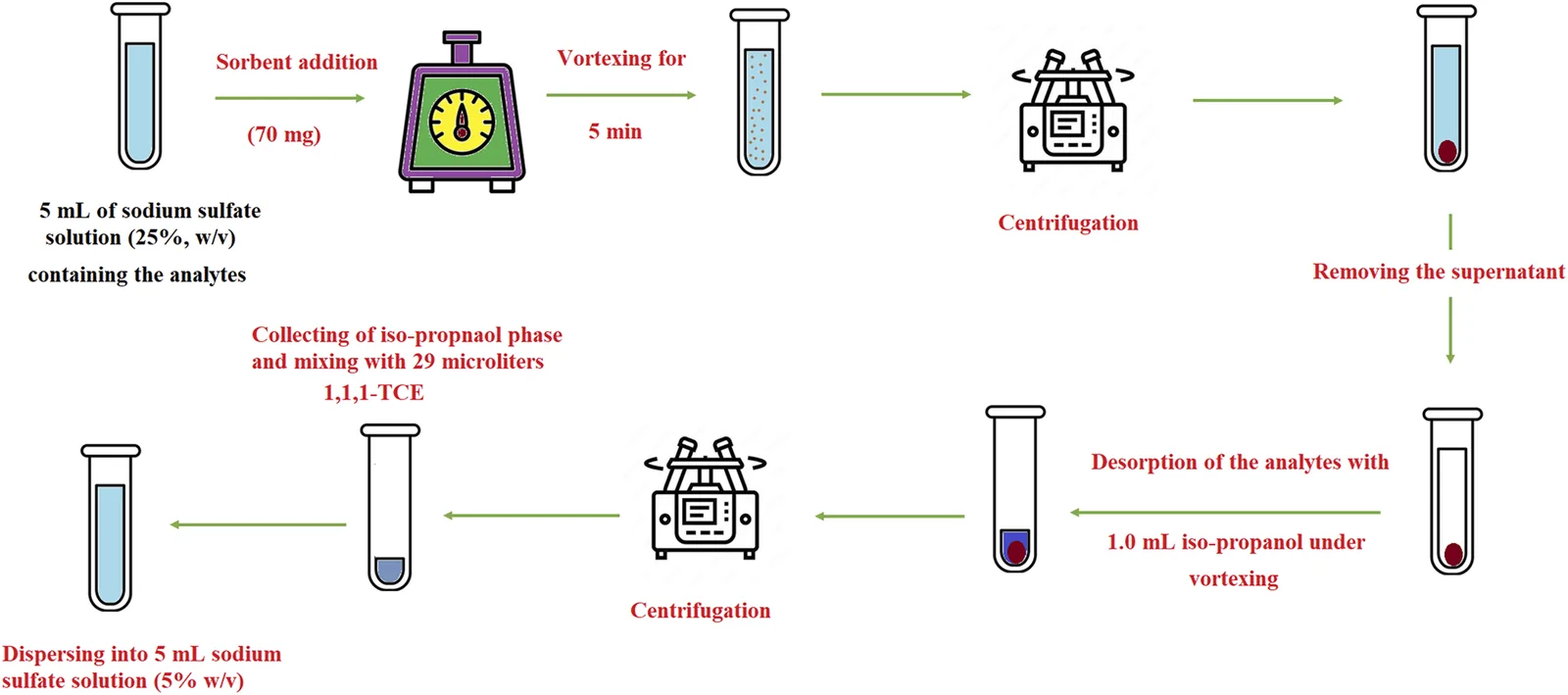

## 1. Introduction

The extensive application of pesticides in modern agriculture has led to their widespread occurrence in food products and environmental matrices. Among various food commodities, grape and grape-derived beverages are particularly susceptible to pesticides contamination due to intensive vineyard treatments and the physicochemical properties of grape, which facilitate adsorption and penetration of pesticides. The presence of trace levels of pesticide residues in grape juice raises serious concerns regarding food safety and public health, necessitating the development of sensitive and reliable analytical methods for their determination.^1,2^

Haloxyfop-R-methyl, oxadiazon, diniconazole, tebuconazole, and difenoconazole are commonly used herbicides and fungicides in viticulture. These compounds belong to different chemical classes having relatively hydrophobic structures and low water solubility, which complicate their direct analysis in aqueous matrices.^3,4^ Moreover, the complex composition of grape juice, rich in sugars, organic acids, pigments, and phenolic compounds, introduces significant matrix effect that adversely affects chromatographic analysis and detector response. Therefore, efficient sample preparation and matrix cleanup steps are essential before instrumental determination.^5–7^ Dispersive micro solid phase extraction (DµSPE) has emerged as an effective sample preparation technique due to its simplicity, low solvent consumption, and high extraction efficiency.^8,9^ When combined with dispersive liquid–liquid microextraction (DLLME), the resulting hybrid approach benefits from enhanced enrichment factors (EFs) and improved matrix removal. The DµSPE-DLLME coupling is particularly attractive for the extraction of hydrophobic pesticides from complex aqueous matrices.^10,11^ Gas chromatography coupled with flame ionization detection (GC-FID) remains a robust and cost-effective analytical technique for the routine determination of such compounds, especially in the laboratories where mass spectrometric detection is not readily available. In recent years, polymeric sorbents have attracted considerable attention in sample preparation procedures owing to their tunable structures, chemical stability, and abundant functional groups. Amide-linked organic networks, in particular, offer strong hydrogen-bonding interactions and π–π stacking capabilities, which enhance their affinity toward aromatic and heterocyclic pesticides. These materials can be synthesized through straightforward condensation reactions, resulting in three-dimensional cross-linked structures with high adsorption capacity.^12,13^

Despite the growing number of studies on DµSPE-DLLME methods and sorbents, the development of new sorbent materials with improved selectivity and extraction performance remains a significant challenge. In particular, there is still a need for the sorbents with tailored functional groups and structures that can provide stronger interactions with hydrophobic pesticide molecules. In our previous studies, similar extraction strategies have been employed using different sorbents; however, the design and application of amide-based porous polymers for this purpose have not yet been fully explored. Therefore, the present work aims to introduce a newly synthesized amide-based organic polymer as an efficient sorbent, offering enhanced interaction capabilities and improved analytical performance compared to previously reported systems.

In this study, an amide-based organic polymer with was synthesized *via* the polycondensation of 1,3,5-benzenetricarboxylic acid (BTC) and ethylenediamine (EDA) under solvothermal conditions. The synthesized polymer was employed as an efficient sorbent in a DµSPE-DLLME procedure for the extraction and preconcentration of five pesticides from grape juice samples. The proposed DµSPE-DLLME-GC-FID method was systematically optimized and validated, and its applicability was demonstrated through analysis of the selected pesticides in four real grape juice samples.

## 2. Materials and methods

### 2.1. Chemicals and solutions

Analytical standards of haloxyfop-R-methyl, oxadiazon, diniconazole, tebuconazole, and difenoconazole with the purities higher than 98% were obtained from Dr Ehrenstorfer (Augsburg, Germany). For the synthesis of the sorbent, BTC (99%), EDA (99%), and dimethyl formamide (DMF) (99%) were employed as monomers and reaction solvent. Absolute ethanol (99.9%) was used during washing of the synthesized sorbent. All of these chemicals were purchased from Merck (Darmstadt, Germany). The salts used during optimization of the extraction procedure, including potassium chloride (KCl, 99.9%), sodium sulfate (Na_2_SO_4_, 99%), and sodium chloride (NaCl, 99%), as well as sodium hydroxide (NaOH, 99%), and hydrochloric acid (HCl, 37%, w/w) for pH adjustment, were supplied by Fluka (Buchs, Switzerland). Organic solvents such as acetonitrile (ACN, 99.9%), *iso*-propanol (99.8%), methanol (99.9%), and acetone (99.9%), which were used for the analytes desorption in the DµSPE step and as disperser solvents in DLLME, were purchased from Sigma-Aldrich (St. Louis, MO, USA). 1,1,1-Trichloroethane (1,1,1-TCE, 99%), 1,2-dibromoethane (1,2-DBE, 98%), chloroform (99%), and 1,1,2-trichloroethane (1,1,2-TCE, 99.5%) evaluated as the extraction solvents were obtained from Janssen Chimica (Beerse, Belgium). A mixed stock solution of the analytes was prepared by dissolving 10 mg of each pesticide in 10 mL methanol. Working standard solutions were freshly prepared daily by diluting the stock solution with deionized water. Deionized water was supplied by Ghazi Company (Tabriz, Iran) and used throughout the experiments.

### 2.2. Real samples

Four commercial grape juice samples from different brands were purchased from local markets in Tabriz, Iran. Before analysis, each sample was diluted at a 1 : 1 (v/v) ratio with deionized water. The diluted samples were then subjected to the proposed DµSPE-DLLME procedure under optimized conditions.

### 2.3. Instrumentation

Gas chromatographic analyses were carried out using a Shimadzu GC-2014 gas chromatograph (Shimadzu, Kyoto, Japan) equipped with an FID and a split/splitless injection system. Chromatographic separation was achieved on a DB-5MS fused-silica capillary column (30 m × 0.25 mm i.d., 0.25 µm film thickness; Agilent Technologies, Santa Clara, CA, USA). Helium (99.999%), supplied by Gulf Cryo (Dubai, UAE), was used as the carrier gas at a linear velocity of 30 cm s^−1^. Hydrogen for the FID was generated using a Parker Packard hydrogen generator (model A915002NA; Parker Hannifin, Cleveland, OH, USA). The detector temperature was set to 300 °C, while hydrogen and air flow rates were maintained at 30 and 300 mL min^−1^, respectively. The oven temperature programming was started at 60 °C and maintained for 1 min, followed by a rise to 300 °C at a rate of 10 °C min^−1^ and held for 1 min. Additionally, the studied real samples were analyzed using GC-mass spectrometry (MS) system for confirmatory identification of the target analytes, while quantitative analysis was performed using GC-FID data. GC-MS analysis was performed using an Agilent 7890A gas chromatograph coupled to an Agilent 5975C mass selective detector. Separation was achieved on an RTX-5 capillary column (30 m × 0.25 mm i.d. × 0.25 µm film thickness, Restek, Bellefont, PA, USA). Helium (99.999%) was used as the carrier gas at the flow rate of 1 mL min^−1^. Injection port temperature and temperature programming were the same as those used in GC-FID. The mass spectrometer was operated in electron ionization mode at 70 eV. The ion source, transfer line, and injector temperatures were maintained at 250, 260 and 300 °C, respectively. Mass spectra were acquired over the *m*/*z* range of 55–400 at a scan rate of 20 Hz, with a detector voltage of −1700 V. Compounds identification was performed by comparing the mass spectra of the eluted compounds in real samples at the retention times of the analytes with the mass spectra achieved from NIST mass spectral library. Accurate weighing was conducted using an AEL-200 analytical balance (±0.0001 g) and an HR-200 precision balance (±0.01 g) (Shimadzu, Kyoto, Japan). pH of solutions was adjusted using a Metrohm L47 digital pH meter (Metrohm, Herisau, Switzerland). Sample mixing was achieved with a Labinco L46 vortex mixer (Labinco, Breda, the Netherlands). Structural characterization of the synthesized sorbent was performed using complementary techniques. Fourier transform infrared (FTIR) spectrum was recorded with a Bruker FTIR spectrometer (Billerica, MA, USA). X-ray diffraction (XRD) pattern was obtained using a Siemens D500 diffractometer (Siemens, Karlsruhe, Germany). Surface morphology and microstructural features were examined by scanning electron microscopy (SEM) using a Mira 3 microscope (Tescan, Brno, Czech Republic) equipped with an energy-dispersive X-ray (EDX) detector. The specific surface area and pore size distribution were determined by the Brunauer–Emmett–Teller (BET) method using a BELSORP-mini analyzer (MicrotracBEL, Osaka, Japan).

### 2.4. Synthesis of sorbent

The organic polymer was synthesized *via* polycondensation reaction. Briefly, 1.3 g of BTC was dissolved in 50 mL of DMF under continuous stirring. Subsequently, 0.6 mL of EDA was added dropwise to the solution. The reaction mixture was maintained in an oil bath at 130 °C for 3 h, allowing the gradual removal of the condensation by-product (water) and promoting formation of the amide-linked polymeric. After completion of the reaction, the resulting solid was filtered and sequentially washed three times with DMF, absolute ethanol, and deionized water (each 10 mL), respectively, to eliminate unreacted monomers and impurities. The purified material was then dried under vacuum at 50 °C for 2 h before use.

### 2.5. Extraction procedure

A 5.0 mL aliquot of diluted grape juice (see Section 2.2) sample or deionized water spiked with 200 µg L^−1^ of each pesticide was transferred into a 10 mL glass test tube. Subsequently, 25% (w/v) Na_2_SO_4_ was added to the solution, followed by brief vortex mixing to ensure complete homogenization. Next, 70 mg of the sorbent was added to the tube, and the mixture was vortexed for 5 min to promote effective adsorption of the target analytes onto the sorbent surface. The mixture was then centrifuged at 7000 rpm for 5 min. The supernatant aqueous phase was carefully discarded. For the desorption step, 1.0 mL of *iso*-propanol was added directly onto the sorbent, and the mixture was vortexed for 5 min to facilitate the analytes desorption. After centrifugation at 7000 rpm for 5 min, the resulting supernatant was quantitatively transferred into a small glass vial. For the subsequent DLLME procedure, 5.0 mL of deionized water was placed into a 10 mL conical bottom glass test tube, and 5% (w/v) Na_2_SO_4_ was added. The mixture was briefly vortexed to obtain a homogeneous solution (aqueous solution used in DLLME). Meanwhile, 29 µL of 1,1,1-TCE was added to the eluate extract obtained from the DµSPE step and mixed thoroughly. The resulting mixture was rapidly injected into the aqueous phase using a 2 mL syringe, to form a cloudy solution, which indicated effective dispersion and extraction. Phase for formation separation was achieved by centrifugation at 7000 rpm for 5 min. Finally, 1.0 µL of the settled organic phase at the bottom of the tube was withdrawn using a micro syringe and injected into the GC-FID system for analysis. A schematic illustration of the overall extraction procedure is presented in Fig. 1.

**Fig. 1:**
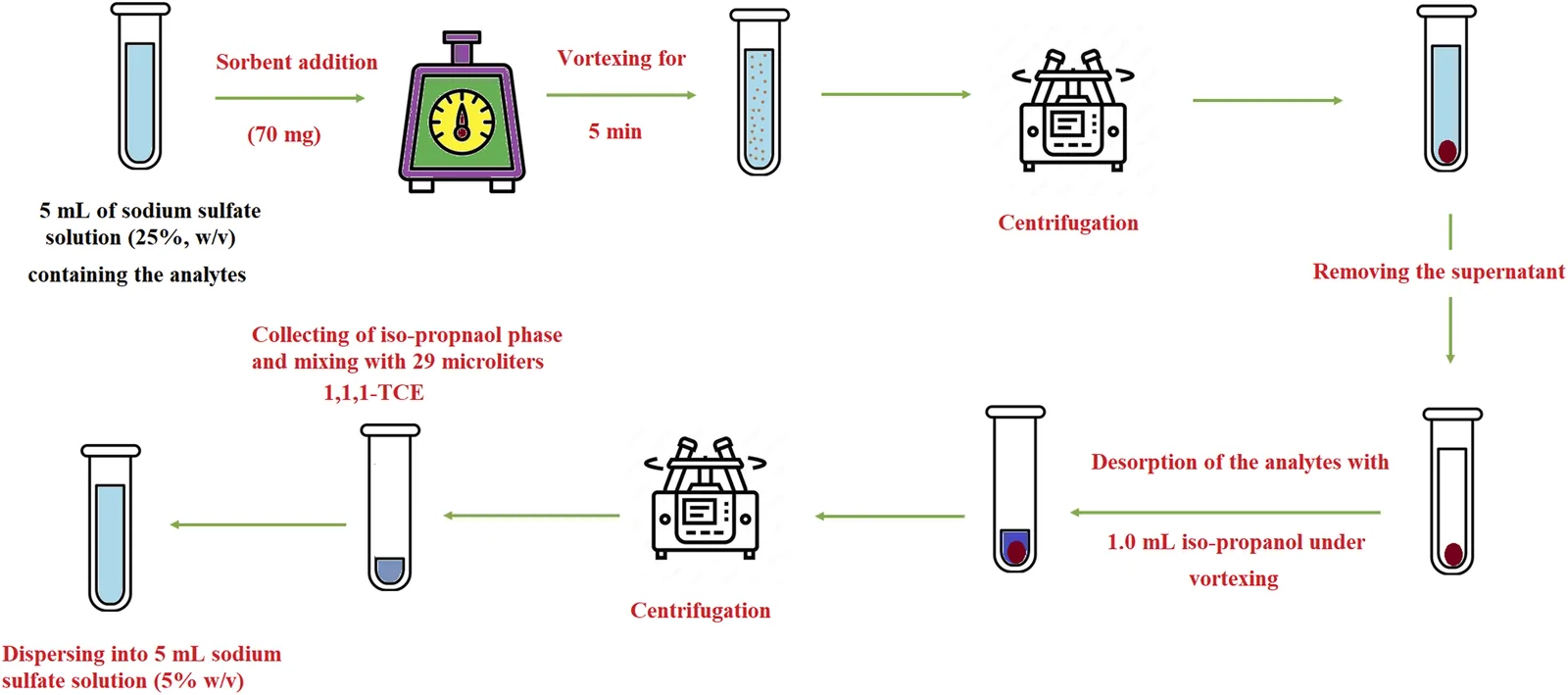
Extraction procedure.

### 2.6. Enrichment factor and extraction recovery calculation

Enrichment factor (EF) is defined as the ratio of analyte concentration measured in the final sedimented extract (*C*_sed_) and its original concentration in the aqueous solution (*C*_0_). This parameter is widely employed to assess the performance of an extraction method, as it reflects the extent to which analyte is enriched during the extraction process and its partitioning behavior between organic extractive phase and aqueous solution. The mathematical expression of EF is provided in eqn (1).1
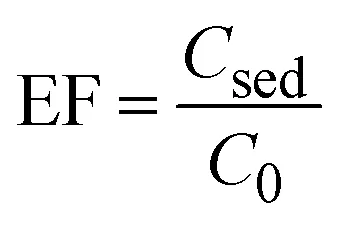


Extraction recovery (ER) represents the proportion of the total analyte initially present in the aqueous phase (*n*_0_) that is successfully transferred into the extractive phase (*n*_sed_). This value provides a direct measure of the extraction capability of the method. The corresponding relationship is described in eqn (2).2
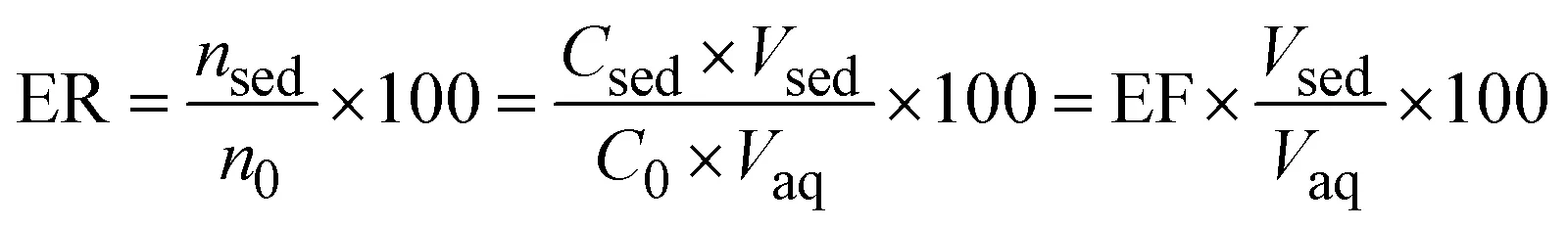
In this equation, *V*_aq_ denotes volume of the aqueous phase, whereas *V*_sed_ refers to volume of the sedimented organic phase obtained after extraction.

### 2.7. Evaluation of sorbent reusability

In order to evaluate the sorbent re-suability, 5 mL of Na_2_SO_4_ solutions (25% w/v) spiked at two concentrations of 200 and 400 µg L^−1^ (each analyte) was extracted by 70 mg of sorbent under vortexing for 5 min. Then, the adsorbed analytes were eluted by 1.0 mL of *iso*-propanol (under vortexing for 5 min). After centrifugation, *iso*-propanol was separated and mixed with 29 µL of 1,1,1-TCE. This mixture was injected into 5 mL of Na_2_SO_4_ solutions (5% w/v). The cloudy solution was centrifuged and the sedimented phase was used for the analysis by GC-MS. After each cycle, the sorbent particles were collected and contacted with 5 mL ethanol under vortexing for 5 min. Then, the mixture was centrifuged and dried in an oven adjusted to 60 °C.

## 3. Results and discussion

### 3.1. Characterization of sorbent

The chemical structure of the synthesized sorbent was investigated by FTIR spectrum, as illustrated in Fig. 2a. The broad absorption band observed in the region of 3200–3500 cm^−1^ is attributed to the overlapping stretching vibrations of –NH and –OH groups, indicating the formation of amide functional group and the presence of hydrogen bonding within the polymer. The appearance of a strong band around 1650–1680 cm^−1^ corresponds to the carbonyl (C=O stretching) vibration, confirming successful polyamidation between BTC and EDA. Additionally, the band observed near 1540–1560 cm^−1^ is assigned to another amide band, arising from N–H bending coupled with C–N stretching vibrations. Characteristic peaks in the range of 1200–1350 cm^−1^ are related to C–N stretching, further supporting the formation of an amide-linked polymer. The presence of aromatic ring originating from BTC is evidenced by absorption bands in the region of 1450–1600 cm^−1^. Overall, the FTIR spectrum confirms the successful formation of the amide-based polymer.

**Fig. 2:**
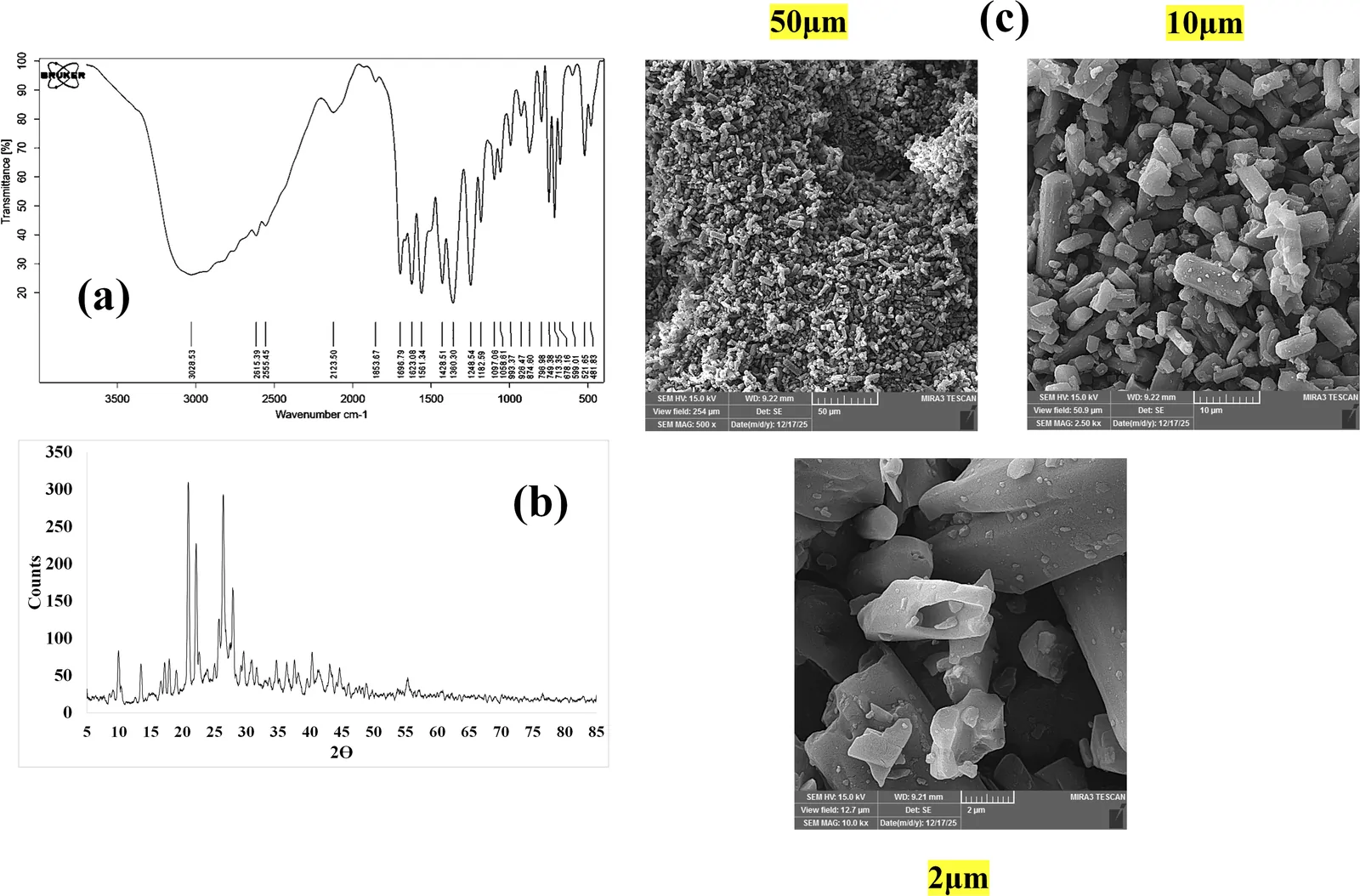
FTIR spectrum (a), XRD pattern (b), and SEM images (c).

The crystalline nature of the synthesized sorbent was examined by XRD, as shown in Fig. 2b. The diffraction pattern displays a broad and poorly defined features with no district long range order, indicating a predominantly amorphous structure. This amorphous character is typical for the polymer formed *via* covalent polycondensation reactions. The lack of long-range crystallinity can be attributed to the random crosslinking between BTC and EDA, which leads to a highly disordered polymeric network. Such an amorphous morphology is advantageous for extraction applications, as it generally provides a high density of accessible adsorption sites for the target analytes.

The surface morphology and microstructure of the synthesized sorbent were investigated using SEM images obtained at different magnifications (2, 10, and 50 µm), as presented in Fig. 2c. At lower magnification (50 µm), the sorbent exhibits an irregular and aggregated morphology composed of interconnected particles. Increasing the magnification to 10 µm reveals a rough surface with fragmented and rod-like structures, suggesting the formation of a network with non-uniform particles size. At the highest magnification (2 µm), the material shows angular and layered features with visible voids and inter particle spaces, which can facilitate mass transfer and enhance adsorption efficiency during the extraction process. The observed morphology confirms the formation of a heterogeneous and polymeric framework suitable for extraction.

The elemental composition of the synthesized sorbent was analyzed by EDX, and the corresponding spectrum is shown in Fig. S1. The EDX results confirm the presence of carbon, nitrogen, and oxygen as the main constituent elements. The presence of nitrogen provides strong evidence for the incorporation of amide linkages, while the high oxygen content is consistent with the carboxyl-derived functional groups originating from BTC. The absence of any extraneous elements indicates the high purity of the synthesized material and the successful removal of unreacted precursors. To confirm the porous structure of the sorbent, it was analyzed by BET and the obtained nitrogen adsorption/desorption isotherm, BET plot, and BHJ (ads) are obtained. The nitrogen adsorption/desorption isotherm shows a Type IV isotherm, characterizing the mesoporous nature of the sorbent. The sharp increase at high relative pressure indicates capillary condensation, with a maximum adsorption volume of 258 cm^3^ g^−1^. To study the specific surface area, the BET model was used to the adsorption data. The high degree of linearity confirms the validity of the BET model for this sample. The pore volume of 0.38 cm^3^ g^−1^ and a BET surface area of 286 m^2^ g^−1^ were determined for the prepared sorbent. The BHJ method was applied to calculate the pore size distribution of the sample. The graph exhibits a sharp peak in the lower mesopore region (below 10 nm), indicating that the majority of the pore volume is contributed by small mesopores. As the pore diameter increases beyond 20 nm, the pore volume drops significantly and remains nearly constant. This confirms the mesoporous nature of the material and is in excellent agreement with the Type IV isotherm. The average pore size was 3.5 nm. It is obvious that the adsorption performance of the sorbent is related to the textural properties of sorbent. The mesoporous structure of sorbent with relatively high surface area, provides sufficient active sites for the analytes. The pore size distribution below 10 nm acceptable total volume facilitates efficiency mass transfer and provides sufficient space for accommodating the adsorbate.

### 3.2. Optimization of extraction parameters

#### 3.2.1. Effect of salt type and concentration in DLLME

In the DLLME step, the presence of salt plays a critical role in improving extraction efficiency by modifying the ionic strength of aqueous phase. Salt addition can reduce the solubility of hydrophobic analytes in water through the salting-out effect, thereby promoting their transfer into the organic extraction phase. However, both the nature and amount of salt can significantly influence analytes distribution; therefore, these parameters must be carefully optimized. To evaluate the effect of salt type, three commonly used inorganic salts, namely NaCl, KCl, and Na_2_SO_4,_ were individually added to the aqueous phase at a fixed concentration of 1.0 mol L^−1^. The obtained results were compared with those of salt-free solution. The efficiency of the DLLME process under these conditions was assessed based on the peak area of the target pesticides obtained by GC-FID. As illustrated in Fig. 3a, the addition of salt generally enhances extraction efficiency compared to the saltless condition. Among the investigated salts, Na_2_SO_4_ provides the highest analytical signals for all analytes, indicating superior extraction performance. This behavior can be attributed to the stronger salting-out capability of sulfate ions, which more effectively decreases analytes solubility in the aqueous phase and facilitates their migration into the organic extractant. Consequently, Na_2_SO_4_ was selected as the optimal salt for the subsequent experiments.

**Fig. 3:**
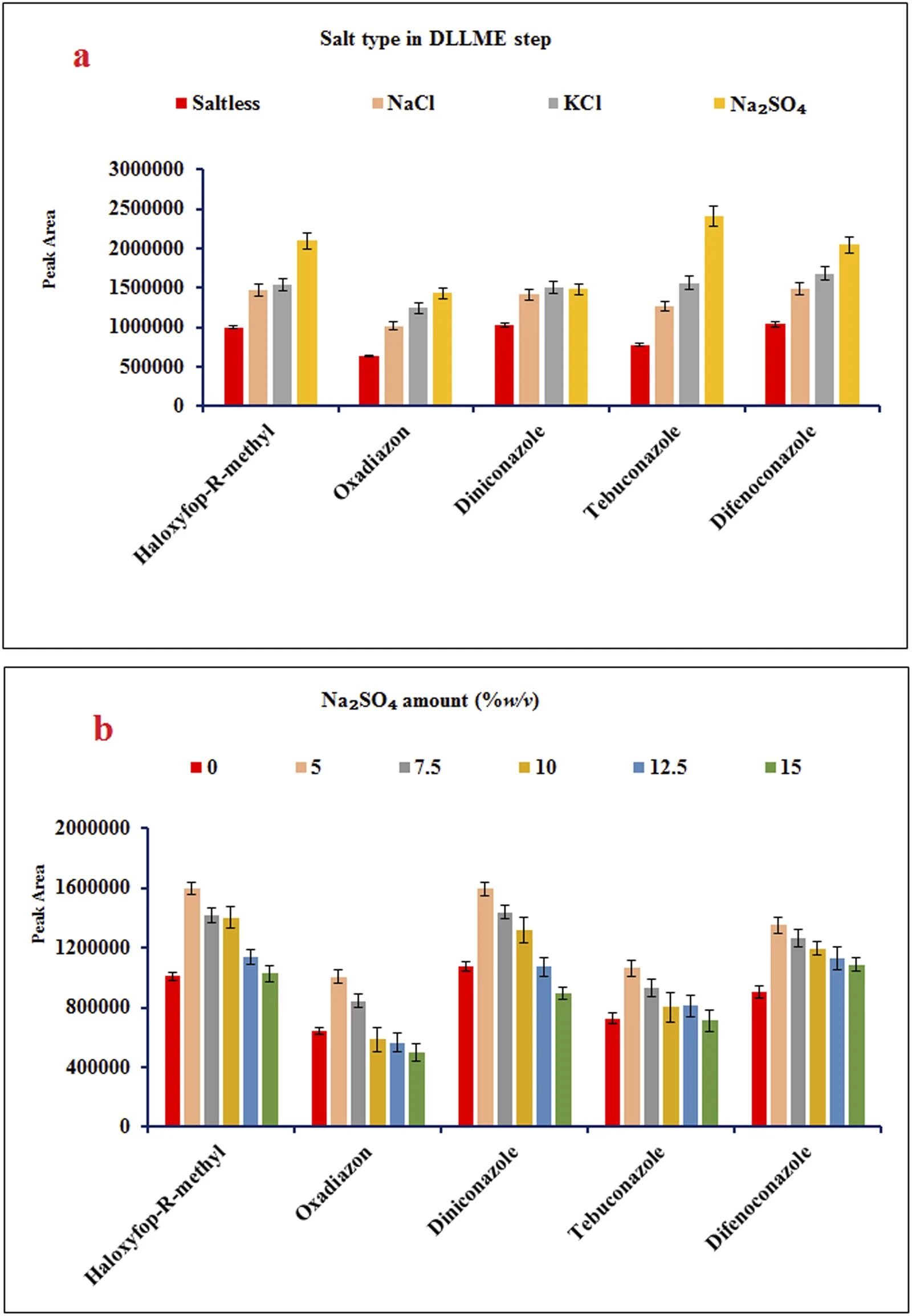
Optimization of salt type (a) and concentration (b) in DLLME step. DLLME conditions: aqueous phase, 5 mL deionized water without salt addition and pH adjustment; disperser solvent, 1 mL of ACN; extraction solvent, 1,2-DBE with a volume of 30 µL; and centrifugation rate and time, 7000 rpm and 5 min, respectively. Error bars depict the minimum and maximum values of three repeated determinations.

Following the selection of the appropriate salt, concentration of Na_2_SO_4_ was optimized to achieve maximum extraction efficiency. For this purpose, Na_2_SO_4_ was added to the aqueous phase at different levels of 0, 5.0, 7.5, 10.0, 12.5, and 15.0% (w/v). The extraction performance under each condition was again evaluated based on the peak areas of the analytes. As shown in Fig. 3b, the peak area values increase markedly upon the addition of salt up to 5.0% (w/v), reflecting an enhanced salting-out effect. However, further increase in the salt concentration leads to a slight decline in extraction efficiency. This phenomenon can be explained by increased solution viscosity and hindered dispersion of the extraction solvent at higher salt contents, which negatively affect mass transfer of the analytes. Accordingly, 5.0% (w/v) Na_2_SO_4_ was selected as the optimal salt concentration for the DLLME procedure.

#### 3.2.2. Selection of disperser solvent type and volume in DLLME

In DLLME, disperser solvent plays a crucial role in achieving rapid and uniform dispersion of extraction solvent throughout the aqueous phase. An appropriate disperser solvent should be miscible with both the aqueous solution and extraction solvent to facilitate the formation of a stable cloudy system, which directly affects mass transfer and extraction efficiency. Therefore, the type and volume of the disperser solvent were carefully optimized. To investigate the effect of disperser solvent type, four commonly used organic solvents, including *iso*-propanol, ACN, acetone, and methanol, were evaluated. For this purpose, a constant disperser solvent volume of 1.0 mL was employed, and all other experimental parameters were kept unchanged. The extraction efficiency under each condition was assessed based on the peak areas of the target analytes. As depicted in Fig. 4a, *iso*-propanol produces the highest analytical signal values among the tested solvents. This enhanced performance can be attributed to its suitable polarity and excellent miscibility with both aqueous phase and extraction solvent, leading to more efficient dispersion and improved analytes transfer. Accordingly, *iso*-propanol was selected as the optimal disperser solvent.

**Fig. 4:**
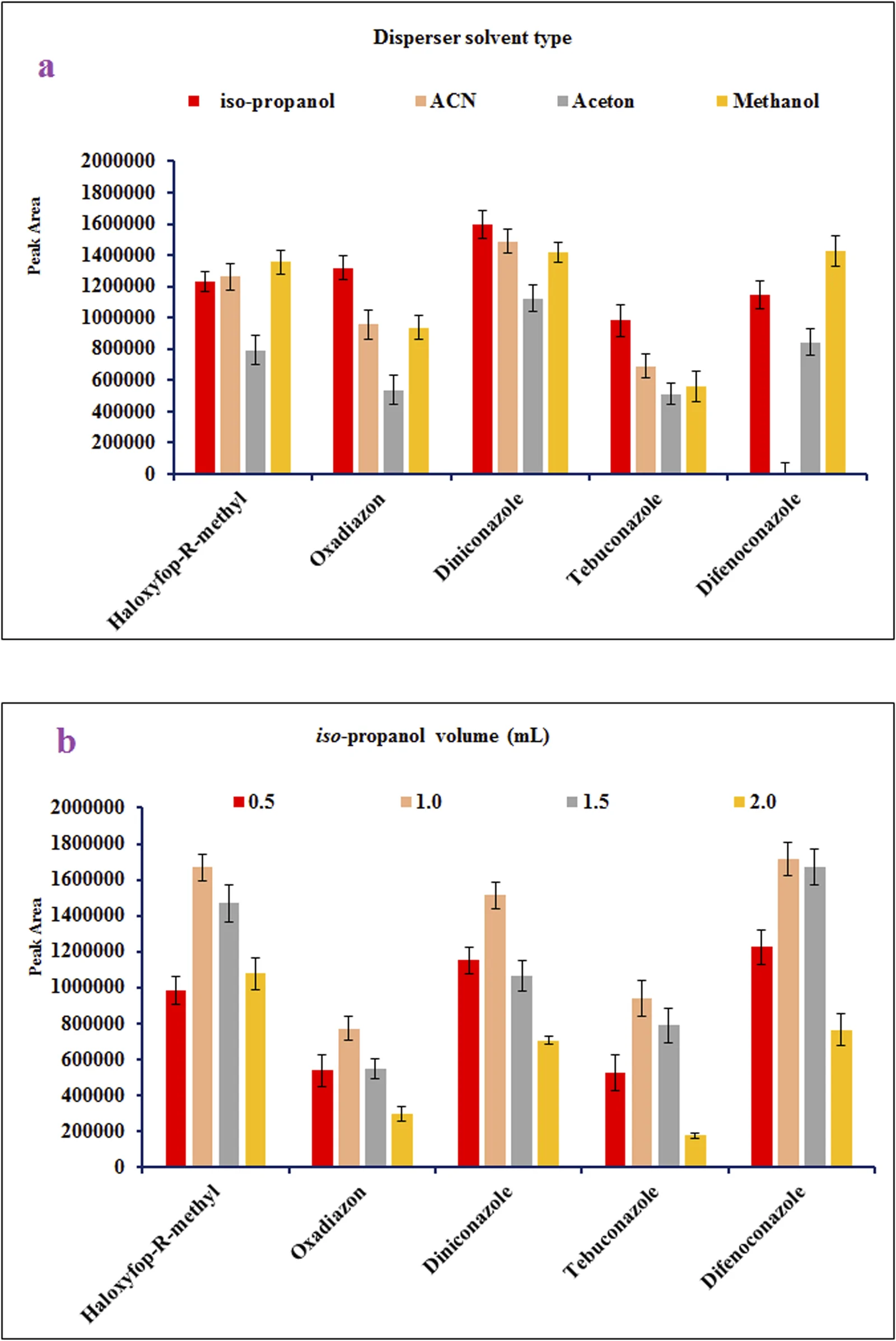
Optimization of disperser solvent type (a) and volume (b) in DLLME step. Extraction conditions were identical to those implemented in Fig. 3, except for the utilization of 5% (w/v) Na_2_SO_4_.

Following the selection of the appropriate disperser solvent, the influence of its volume on the DLLME performance was examined. The volumes of 0.5, 1.0, 1.5, and 2.0 mL were investigated while maintaining all other conditions constant. The results, shown in Fig. 4b, demonstrate that the peak area values increase with increasing disperser solvent volume up to 1.0 mL, indicating enhanced dispersion and extraction efficiency. However, further volume increase results in decreased peak area values, likely due to dilution effect or partial solubilization of the analytes in the aqueous phase. Therefore, 1.0 mL of *iso*-propanol was selected as the optimal disperser solvent volume for the DLLME procedure.

#### 3.2.3. Selection of extraction solvent type and volume in DLLME

The nature of the extraction solvent is one of the most influential parameters in DLLME, as it governs the partitioning of analytes between aqueous and organic phases, stability of the cloudy system, and efficiency of phase separation. An ideal extraction solvent should exhibit low solubility in water, high affinity toward the target analytes, and an appropriate density to allow easy collection of the sedimented phase after centrifuging. Both the type and volume of the extraction solvent were systematically optimized. To investigate the effect of extraction solvent type, four high density organic solvents namely 1,2-DBE, 1,1,1-TCE, 1,1,2-TCE, and chloroform were evaluated. In order to ensure a fair comparison among the tested solvents, the volume of each solvent was individually adjusted so that an approximately constant sedimented organic phase volume of 10 ± 0.5 µL was obtained after centrifugation. Accordingly, the solvent volumes of 30, 29, 27, and 48 µL were used for 1,2-DBE, 1,1,1-TCE, 1,1,2-TCE, and chloroform, respectively. The extraction efficiencies were compared based on the peak area of the analytes. As illustrated in Fig. 5a, 1,1,1-TCE provides superior extraction performance for the majority of the target pesticides, which can be attributed to its favorable physicochemical properties and stronger interactions with the studied compounds. Consequently, 1,1,1-TCE was selected as the optimal extraction solvent for the further optimization.

**Fig. 5:**
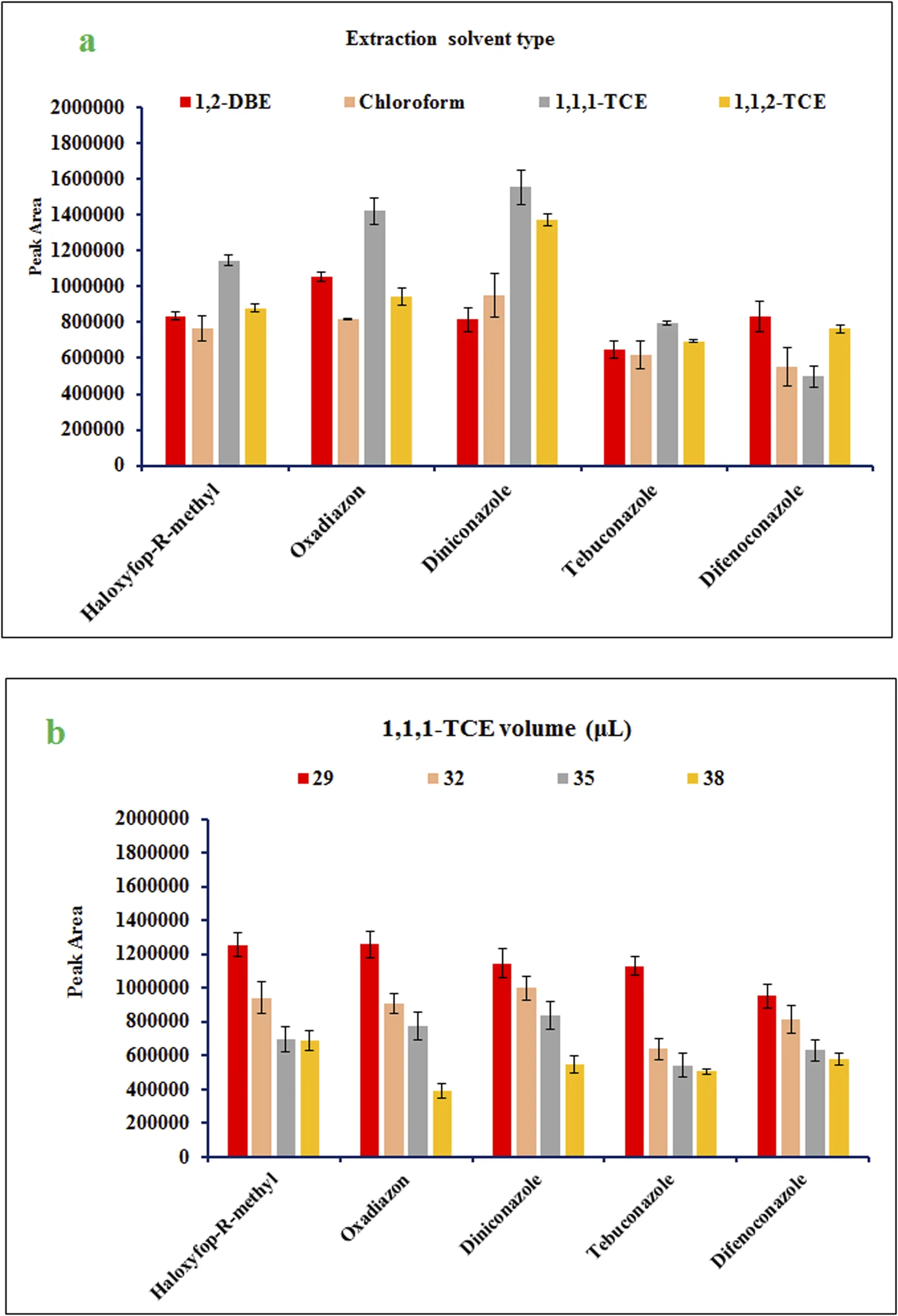
Selection of extraction solvent type (a) and volume (b) in DLLME step. The extraction conditions were the same as those shown in Fig. 4, except that 1.0 mL *iso*-propanol was used as the disperser solvent.

Following the selection of extraction solvent, the influence of 1,1,1-TCE volume on DLLME efficiency was examined. The volumes of 29, 32, 35, and 38 µL were evaluated, which resulted in the formation of approximately 10, 13, 15, and 18 µL the of sedimented organic phase, respectively. The corresponding results, presented in Fig. 5b, indicate that 29 µL of the extraction solvent produces high analytical signals. However, further increase leads to a decrease in extraction efficiency, which can be explained by dilution of the analytes in a larger volume of the sedimented phase, resulting in lower enrichment. Based on these observations, 29 µL of 1,1,1-TCE was considered optimal for achieving maximum enrichment and sensitivity.

#### 3.2.4. Effect of aqueous solution pH in DLLME

The pH of aqueous phase is a critical parameter in DLLME, as it directly influences the ionization state, chemical stability, and partitioning behavior of analytes between the aqueous and organic phases. Variations in pH can alter the distribution coefficient of the target compounds and, consequently, affects extraction efficiency. Therefore, optimization of pH was considered essential to achieve maximum extraction performance. To evaluate the effect of aqueous phase pH, it was adjusted at 3.0, 5.0, 7.0, 9.0, and 11.0 using appropriate volumes of 0.1 M HCl or NaOH solutions, while all other experimental parameters were kept constant. The extraction efficiency under each condition was assessed based on the peak areas of the target pesticides. As shown in Fig. S2, the peak area values of all analytes increase as the pH is adjusted toward neutral conditions and reach a maximum at pH 7.0. At lower pH values, partial protonation and possible instability of some analytes may reduce their affinity for the organic extraction solvent, whereas at higher pH values, changes in molecular interactions and increased solubility in the aqueous phase can adversely affect extraction efficiency. Consequently, pH 7.0 was selected as the optimal aqueous phase pH for the DLLME procedure and applied in all subsequent experiments.

#### 3.2.5. Optimization of sorbent amount in DµSPE

In the DµSPE step, the amount of sorbent is a key factor that directly affects the adsorption and overall ER of analytes. Insufficient sorbent may lead to incomplete extraction, whereas an excessive amount can cause difficulties in the analytes desorption and unnecessary consumption of materials. Therefore, careful optimization of the sorbent mass is essential for achieving high extraction efficiency and method reproducibility. To determine the optimal amount of the sorbent, different masses of 10, 30, 50, 70, and 100 mg were evaluated under identical conditions. The ER values of the target pesticides were calculated for each sorbent amount. As shown in Fig. 6, increasing the sorbent mass from 10 to 70 mg results in a progressive improvement in ER values for all analytes, reflecting enhanced adsorption capability. However, further increase up to 100 mg does not provide a significant improvement in ERs, indicating that the sorbent adsorption sites are already sufficient at 70 mg. Based on these results, 70 mg of the sorbent was selected as the optimal amount for the DµSPE procedure, balancing high extraction efficiency with minimal material usage.

**Fig. 6:**
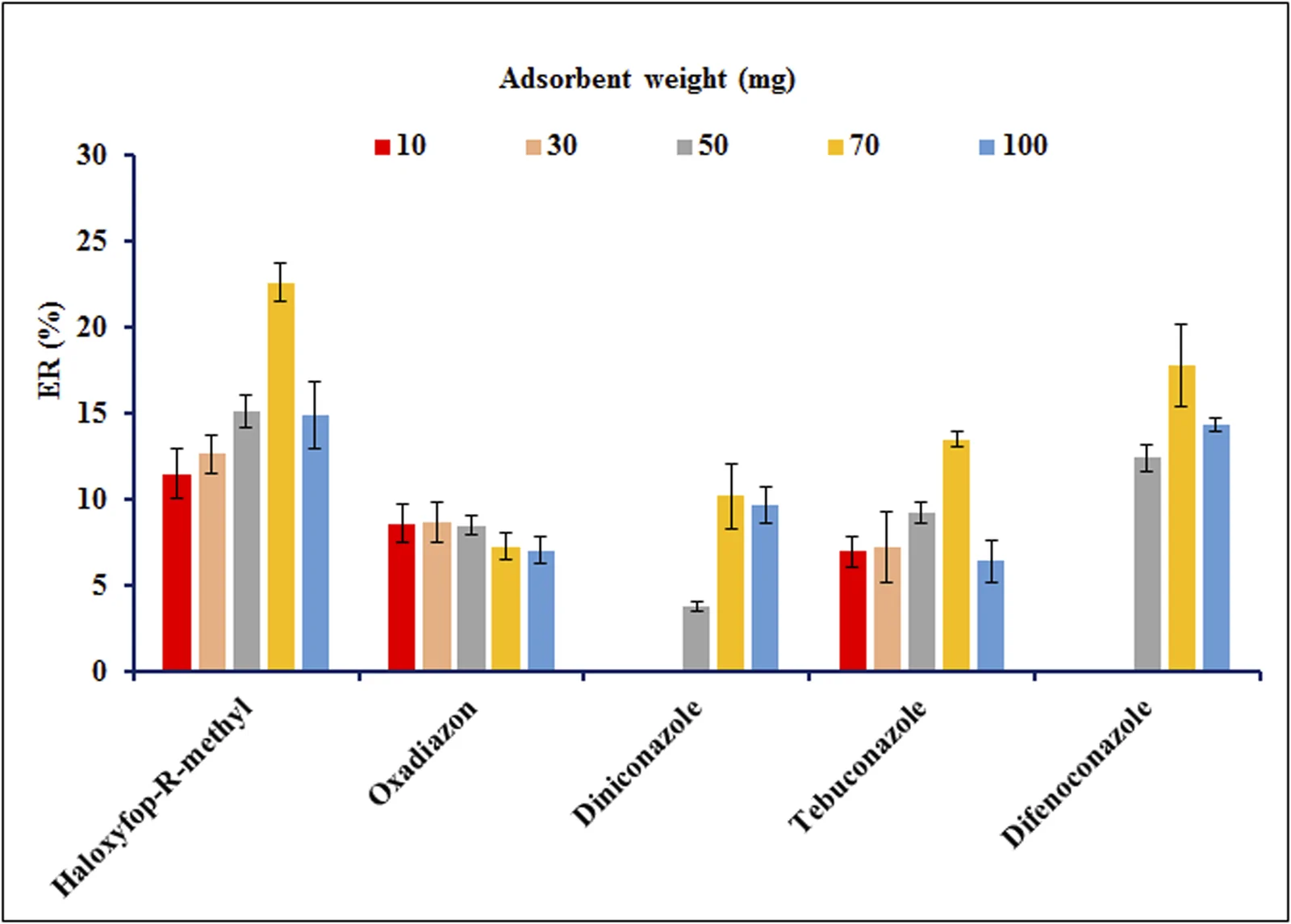
Optimization of sorbent weight. DµSPE conditions: aqueous sample volume, 5 mL of deionized water spiked with 200 µg L^−1^ of each analyte without salt addition and pH adjustment; vortexing time in adsorption step, 5 min; desorption solvent, 1 mL of *iso*-propanol; vortexing time in desorption step, 5 min; and centrifugation rate and time, 7000 rpm and 5 min, respectively. The DLLME conditions used were the same as those shown in Fig. 5, except that 29 µL of 1,1,1-TCE was used as the extraction solvent and pH was 7.0.

#### 3.2.6. Selection of salt type and concentration in DµSPE

The presence of salt in the DµSPE step can influence the extraction efficiency by altering the ionic strength of aqueous sample and enhancing analytes adsorption onto the sorbent surface. Selecting an appropriate salt type and concentration is therefore crucial for maximizing ERs without negatively affecting sorbent performance. To investigate the effect of salt type, three common inorganic salts, namely NaCl, KCl, and Na_2_SO_4_, were added individually to the analytes solution at a fixed concentration of 1.0 mol L^−1^, and their performance were compared with a salt free solution. The extraction efficiency was evaluated based on the ERs of the target pesticides. As shown in Fig. 7a, Na_2_SO_4_ provides the highest ER values across all analytes, indicating that sulfate ions more effectively enhance adsorption of the analytes onto the sorbent surface. Consequently, Na_2_SO_4_ was selected as the optimal salt for the further experiments. Subsequently, the effect of Na_2_SO_4_ concentration on extraction efficiency was examined. The salt concentrations of 0, 10, 20, 25, and 30% (w/v) were tested while maintaining all other conditions constant. The results, presented in Fig. 7b, indicate that concentration of 25% (w/v Na_2_SO_4_ yields the highest ER values for most of the analytes. Increasing the salt concentration beyond this point do not further improve ERs and could potentially hinder adsorption due to increased viscosity of the solution. Therefore, 25% (w/v) Na_2_SO_4_ was selected as the optimal salt concentration for the DµSPE procedure.

**Fig. 7:**
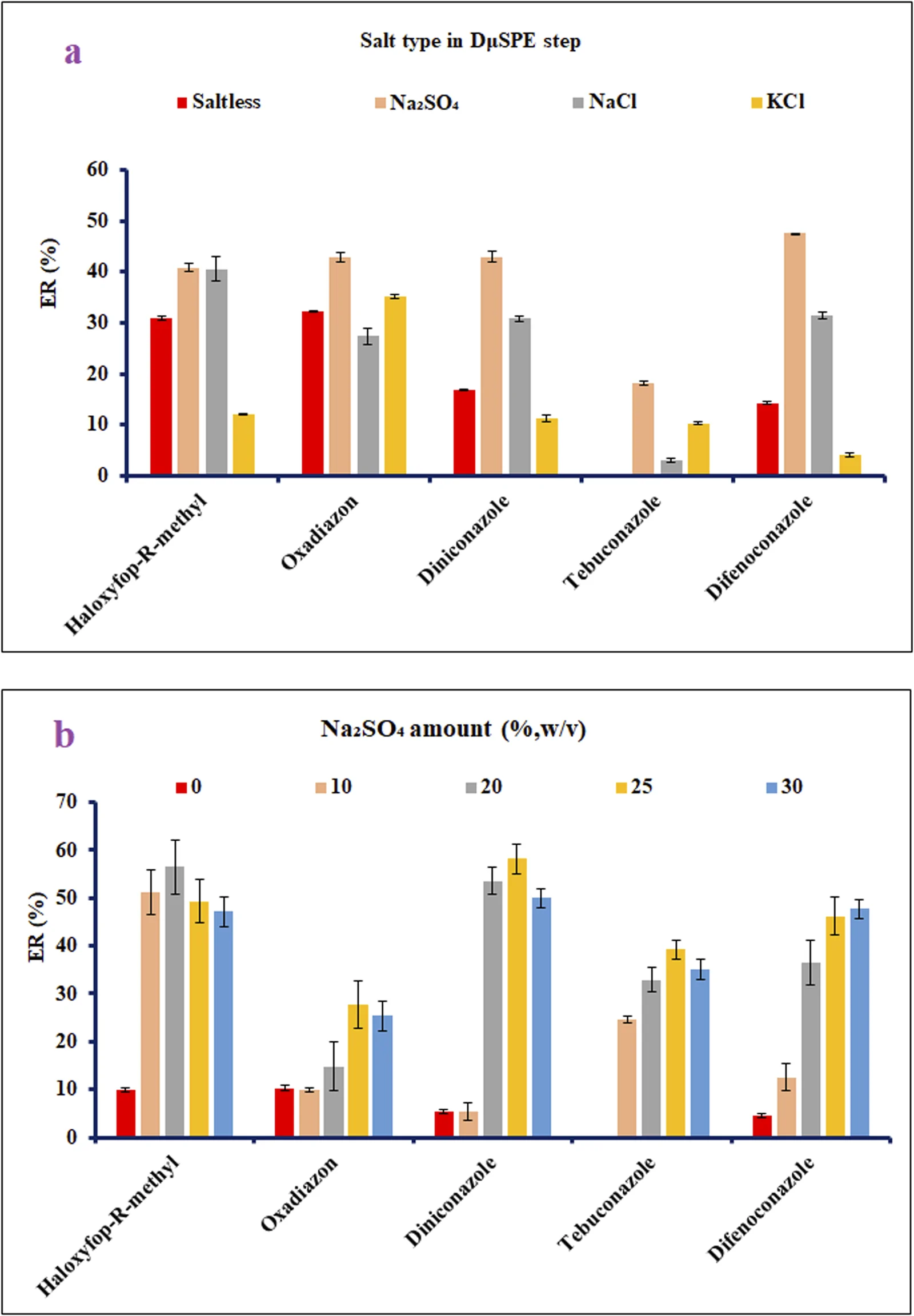
Selection of salt type (a) and concentration (b) in DµSPE step. The extraction conditions were the same as those shown in Fig. 6, except that 70 mg of the sorbent was used.

#### 3.2.7. Effect of vortexing time in adsorption step

The vortexing time during adsorption procedure is a critical factor that directly affects the interactions between the sorbent and analytes. Adequate vortexing ensures thorough contact, facilitating efficient adsorption of the target compounds onto the sorbent surface. However, insufficient or excessive vortexing may result in incomplete adsorption or unnecessary sample handling, respectively. To optimize this parameter, the effect of different vortexing durations (1, 3, 5, and 7 min) on the ERs was investigated while keeping all other experimental conditions constant. The results, presented in Fig. S3, indicate that increasing the vortexing time from 1 to 5 min significantly improves the ER values for all studied pesticides. Extending the vortexing beyond 5 min does not yield further improvement, suggesting that the sorbent and analytes are reached equilibrium state. Therefore, a vortexing time of 5 min was selected as the optimal condition for the adsorption procedure, ensuring efficient analytes extraction without prolonging sample handling.

#### 3.2.8. Selection of desorption solvent type and volume in DµSPE

The choice of desorption solvent in the DµSPE procedure is critical for efficiently recovering analytes from the sorbent surface. An ideal desorption solvent should be compatible with both the sorbent and subsequent DLLME step, ensuring complete analytes releasing while maintaining method consistency. To determine the optimum solvent type, four common organic solvents, *iso*-propanol, ACN, acetone, and methanol, were tested. The ER values of the target pesticides were evaluated while the other conditions were kept constant. As shown in Fig. S4a, *iso*-propanol provides the highest ER values, demonstrating superior ability to desorb analytes from the sorbent surface. After selecting the appropriate solvent, the effect of desorption solvent volume was investigated. The volumes of 0.5, 1.0, 1.5, and 2.0 mL were tested while keeping all other parameters constant. The results, presented in Fig. S4b, indicate that 1.0 mL of *iso*-propanol is sufficient to achieve quantitative desorption for all analytes. Increasing the solvent volume beyond this value does not lead to further improvement, confirming that the analytes are fully eluted by this volume. It is noteworthy that the desorption solvent selected for DµSPE (1.0 mL *iso*-propanol) is identical to the disperser solvent used in the DLLME step, as discussed in Section 3.2.2. This compatibility ensures complete integration of the two extraction steps and consistent analytes transfer throughout the procedure, contributing to the robustness and efficiency of the combined DµSPE–DLLME method.

#### 3.2.9. Effect of vortexing time in desorption step of DµSPE

The vortexing time during the desorption step of DµSPE is an important factor influencing efficiency of the analytes releasing from the sorbent surface into the desorption solvent. Proper vortexing ensures complete interactions between the analytes and solvent, leading to quantitative recovery of the analytes, while insufficient or excessive vortexing can result in incomplete desorption or unnecessary sample handling. To optimize this parameter, vortexing times of 1, 3, 5, and 7 min were tested under identical conditions. The ER values of the target pesticides was used as the evaluation criterion. As presented in Fig. S5, the ER values increase with increasing vortexing time and reach to maximum at 5 min. Extending the vortexing beyond this duration does not provide additional improvement, indicating that complete desorption was achieved. Therefore, a vortexing time of 5 min was selected as the optimal condition for the desorption step, ensuring efficient and reproducible analytes recovery without prolonging the procedure.

### 3.3. Approach validation

In this section, several analytical figures of merit, including coefficient of determination (*r*^2^), limit of detection (LOD), limit of quantification (LOQ), relative standard deviation (RSD), linear range (LR), EF, and ER were employed to validate the proposed method. The corresponding validation results are summarized in Table 1. The LODs and LOQs, calculated at signal-to-noise ratios of 3 and 10, were found to be in the ranges of 2.00–2.58 and 6.60–8.51 µg L^−1^, respectively. The calibration curves for all target analytes showed good linearity over a wide LRs, with *r*^2^ values ranging from 0.9971 to 0.9990. At a concentration level of 200 µg L^−1^, the intra-day precision (*n* = 5) expressed as RSD ranged from 1.14 to 3.65%, while the inter-day precision (*n* = 4) was within 2.13–4.42%, demonstrating satisfactory repeatability and intermediate precision. The EF values, ranging from 365 to 495, confirm the strong enrichment capability of the developed extraction procedure. Moreover, the ER values between 73 and 99%, indicate efficient transfer of the analytes from the aqueous phase to the final organic extract.

**Table 1:** Quantitative features of the developed DµSPE- DLLME- GC-FID method

Analyte	LOD^a^	LOQ^b^	LR^c^	*r* ^2^ d	RSD^e^ % at the concentrations of				EF ± SD^f^	ER ± SD^g^
					200 µg L^−1^		500 µg L^−1^			
					Intra-day	Inter-day	Intra-day	Inter-day		
Haloxyfop-r-methyl	2.45	8.08	8.08–6000	0.9985	1.53	2.13	0.84	2.06	495 ± 10	99 ± 2
Oxadiazon	2.31	7.62	7.62–6000	0.9978	3.65	4.42	2.37	3.20	465 ± 20	93 ± 4
Diniconazole	2.00	6.60	6.60–6000	0.9988	1.14	2.73	1.03	2.31	365 ± 10	73 ± 2
Tebuconazole	2.22	7.32	7.32–6000	0.9971	1.99	2.16	1.28	2.07	390 ± 10	78 ± 2
Difenoconazole	2.58	8.51	8.51–6000	0.9990	3.42	3.68	1.26	2.96	480 ± 15	96 ± 3

a Limit of detection (S/N = 3) (µg L^−1^).

b Limit of quantification (S/N = 10) (µg L^−1^).

c Linear range (µg L^−1^).

d Coefficient of determination.

e Relative standard deviation for intra- (*n* = 5) and inter-day (*n* = 4) precisions.

f Enrichment factor ± standard deviation (*n* = 3).

g Extraction recovery ± standard deviation (*n* = 3).

### 3.4. Reusability of sorbent

The reusability of the synthesized sorbent was evaluated in two concentrations (200 and 400 µg L^−1^ of each analyte) to assess its stability and practical applicability. For this purpose, the sorbent was subjected to five consecutive extractions. Desorption cycles were performed under the optimized DµSPE-DLLME conditions. After each cycle, the sorbent was regenerated and reused without any additional treatment. The obtained results demonstrated that the sorbent maintained comparable extraction performance up to four cycles, with no noticeable decrease in efficiency. However, at the extraction cycles more than 4 times, the sorbent efficiency reduced significantly (≥15%). According to the results in Fig. 8, the RSD values of the analytical signals for the concentration of 200 µg L^−1^ were between 4.1 and 9.0%, and for the concentration of 400 µg L^−1^ were between 6.0 and 14.0%, conforming good repeatability and satisfactory reusability of the sorbent. These findings indicate that the proposed material possesses adequate stability and can be effectively reused for multiple analytical cycles. The slight decrease in extraction efficiency after four cycles cannot be attributed to the instability of the sorbent, as amide-based polymers are known for their high chemical and structural stability, which is well-documented in the literature.^14^ Instead, this behavior is related to a critical methodological limitation of the developed method. In this study, analyte desorption is performed using only 1.0 mL of iso-propanol for 70 mg of sorbent, resulting in a very low solvent-to-solid ratio. This limited volume of iso-propanol is essential to maintain the high enrichment factor and phase separation efficiency of the subsequent DLLME step. Consequently, during each cycle, a fraction of the strongly adsorbed pesticide molecules remains entrapped within the polymer's mesopores, leading to the progressive occupation of a small fraction of active sites. This phenomenon accounts for the slight reduction in extraction efficiency over successive cycles and represents a methodological constraint rather than a structural degradation of the polymer sorbent.

**Fig. 8:**
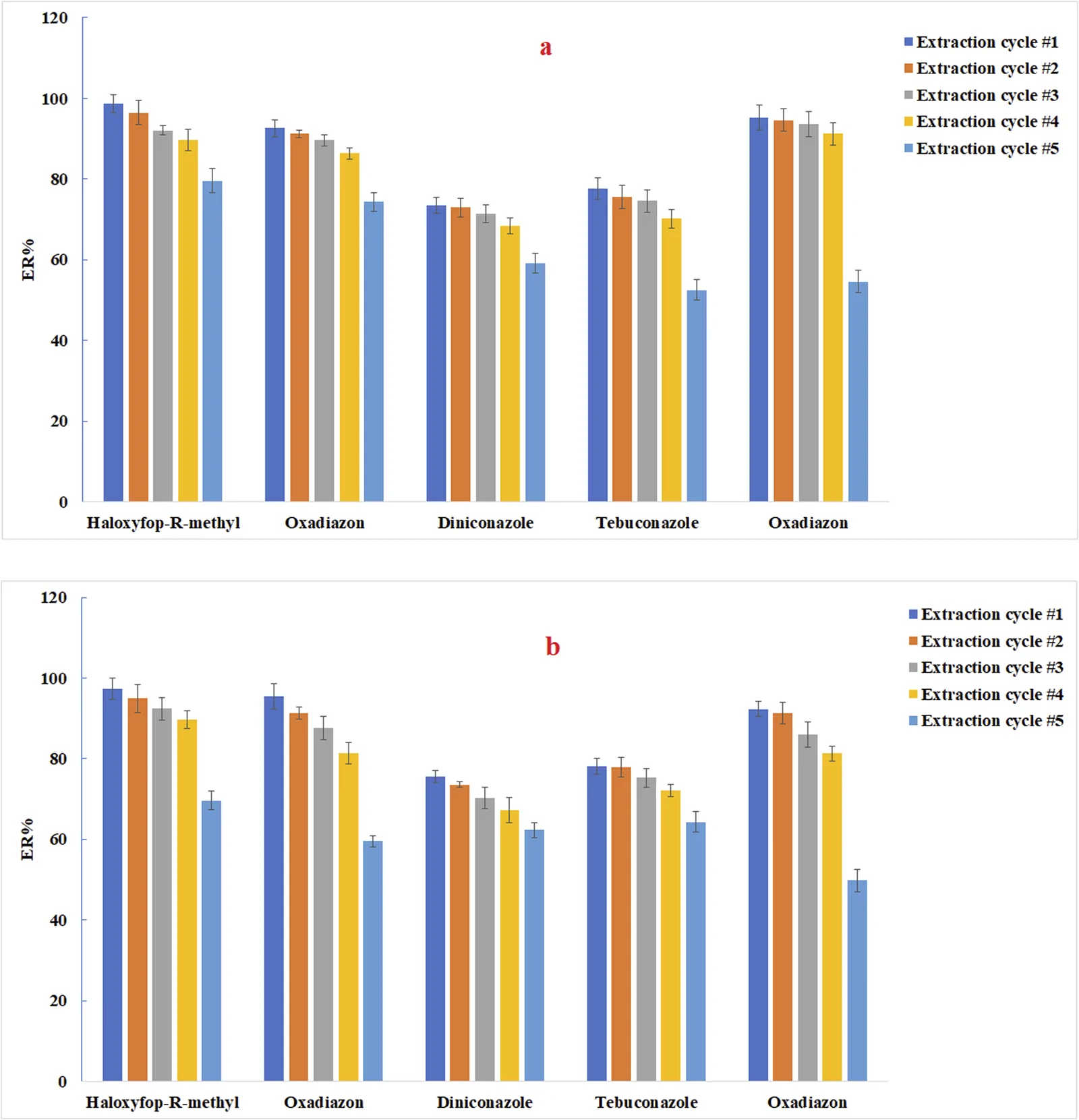
Data of reusability of sorbent in the concentrations of 200 µg L^−1^ (a) and 400 µg L^−1^ (b).

### 3.5. Analysis of real samples and matrix effect study

The applicability of the proposed DµSPE-DLLME-GC-FID method was evaluated by analyzing four grape juice samples. Matrix effect was evaluated by comparison of intercept and slop of the calibration curves obtained using diluted grape juice samples (1 : 1, v/v) and aqueous standard solutions to ensure realistic analytical conditions. Differences of the intercepts and slopes were less than 12% for the mentioned solutions. To minimize matrix complexity, all samples were diluted at a 1 : 1 (v/v) ratio with deionized water before analysis. Also, the possible influence of matrix components on the analytical performance was investigated through relative recovery (RR) experiments, and the results are summarized in Table 2. The grape juice samples were spiked with the target pesticides at the concentration levels of 200 and 500 µg L^−1^ of each pesticide and analyzed under the optimized conditions. The spiking levels (200 and 500 µg L^−1^ of each pesticide) were selected based on preliminary experiments and reported residue levels in the literature. These concentrations fall within the LR of the method and are representative of realistic contamination scenarios in grape juice samples, supporting the fit-for-purpose validation of the method. At the spiking level of 200 µg L^−1^, the RR values ranged from 89–118% across all analytes and samples. Similarly, for the 500 µg L^−1^ spiking level, RR values were obtained in the range of 88–119%. In all cases, the RRs fell within the acceptable range of 80–120%, indicating that matrix constituents of grape juices do not significantly affect the extraction efficiency or chromatographic determination of the analytes. The similarity of analytical responses obtained in matrix-matched and aqueous standard solutions further confirms the negligible matrix effect under the proposed conditions. These results demonstrate that the developed method exhibits good accuracy against matrix effect, confirming its suitability for the determination of the selected pesticides in complex grape juice matrices. Among the analyzed samples, the grape juices #1 and #4 exhibited detectable levels of oxadiazon with the concentrations of 82 ± 3 and 77 ± 3 µg L^−1^ (*n* = 3), respectively. Also, grape juice #2 exhibited detectable levels of haloxyfop-R-methyl, oxadiazon, diniconazole, and tebuconazole, with the concentrations of 18 ± 1, 42 ± 1, 59 ± 1, and 68 ± 2 µg L^−1^ (*n* = 3), respectively. In contrast, none of the target pesticides were detected in grape juice #3, indicating that their concentration is below than LODs of the approach. The mass spectra and GC-MS chromatograms of the analyzed real samples are presented in Fig. S5, clearly illustrating the characteristic ion fragments corresponding to target analytes. GC-MS data verify the results of GC-FID for the present of the analytes in the above-mentioned samples.

**Table 2:** Study of matrix effect in the grape juice samples spiked at different concentrations. All samples were diluted at a 1 : 1 (v/v) ratio with deionized water before analysis

Analyte	Mean relative recovery ± standard deviation (*n* = 3)			
	Grape juice #1	Grape juice #2	Grape juice #3	Grape juice #4
**The samples were spiked with each analyte at a concentration of 200 µg L** ^**−1**^				
Haloxyfop-r-methyl	100 ± 1	100 ± 1	115 ± 2	109 ± 2
Oxadiazon	93 ± 3	108 ± 4	109 ± 4	100 ± 4
Diniconazole	112 ± 1	92 ± 1	118 ± 1	109 ± 1
Tebuconazole	89 ± 2	115 ± 2	107 ± 2	104 ± 2
Difenoconazole	97 ± 3	108 ± 4	92 ± 3	101 ± 3

**The samples were spiked with each analyte at a concentration of 500 µg L** ^**−1**^				
Haloxyfop-r-methyl	97 ± 2	91 ± 2	103 ± 2	107 ± 2
Oxadiazon	93 ± 3	88 ± 3	95 ± 3	98 ± 3
Diniconazole	95 ± 2	94 ± 2	104 ± 2	102 ± 2
Tebuconazole	96 ± 2	115 ± 2	102 ± 2	104 ± 2
Difenoconazole	99 ± 3	98 ± 3	105 ± 4	119 ± 4

### 3.6. Comparison with other reported methods

The analytical performance of the developed DµSPE-DLLME-GC-FID method was compared with several previously reported extraction and determination techniques for pesticides analysis, as summarized in Table 3. The comparison was conducted in terms of sensitivity, linearity, precision, enrichment capability, and applicability to complex matrices. In comparison with earlier microextraction-based GC–FID methods, such as HS-SDME-GC-FID and DµSPE-DLLME-GC-FID applied to fruit juices, the proposed method provides comparable or lower RSD values and higher EFs, reaching up to 495, while maintaining excellent ERs (73–99%). Although some LC-MS/MS-based methods report lower LODs, they generally require expensive instrumentation, longer analysis times, and more complex sample preparation procedures. Relative to SPE- and µSPE-based methods coupled with HPLC or LC-MS/MS, the present approach offers wider LRs (up to 6000 µg L^−1^) and superior enrichment capability, while relying on simpler and more cost-effective instrumentation. Moreover, the precision of the proposed method (RSD ≤ 2.37%) is notably better than or comparable to those reported for most existing techniques. Compared with previously reported DµSPE-DLLME methods, the developed strategy demonstrates higher EFs and ERs, particularly for haloxyfop-R-methyl, oxadiazon, and difenoconazole. The improved performance can be attributed to the efficient interactions between analytes and the synthesized sorbent, as well as the effective phase dispersion during the DLLME step. Overall, the proposed DµSPE-DLLME-GC-FID method combines high sensitivity, excellent precision, wide LR, and strong enrichment capability with low solvent consumption and instrumental simplicity, making it a competitive and practical alternative for the routine determination of pesticide residues in real samples.

**Table 3:** Comparison of the proposed method with other methods for the determination of the target pesticides

Method	Sample	Analytes	LOD^a^	LOQ^b^	LR^c^	*r* ^2^ d	RSD^e^	EF^f^	ER^g^	Ref.
HS-SDME-GC-FID^h^	Orange juice	Diniconazole	0.85	—	10–100 000	0.994	5.6	346	94	15
		Hexaconazole	0.98	—	10–100 000	0.995	4.8	315	97	
µSPE-HPLC-UV^i^	Water, soil, and wheat	Clodinafop propargyl	2	6	6–700	—	4.6	—	—	16
DMSPE-HPLC-DAD^j^	Water	Haloxyfop-r-methyl	13.6	40.5	50–1000	—	—	≤ 100	—	17
SPE- HPLC-MS-MS^k^	Orange and apple juices	Hexaconazole	0.97	—	5–500	0.9959	1.7	—	—	18
		Tebuconazole	0.95	—	5–500	0.9963	1.2	—	—	
		Diniconazole	1.83	—	5–500	0.9930	2.7	—	—	
DSPE-LC–MS/MS^l^	Rapeseed seed oil	Clodinafop propargyl	1.4	4.8	—	0.999	7.4	—	83.9	19
		Haloxyfop-r-methyl	1.2	3.8	—	0.999	7.6	—	81	
		Difenoconazole	0.6	1.9	—	1	6.5	—	81.2	
		Tebuconazole	0.5	1.6	—	0.999	6.5	—	83.2	
DµSPE-DLLME-GC-FID^m^	Orange and mango juices	Haloxyfop-r-methyl	1.25	4.13	4.13–2000	0.996	7.5	375	75	20
		Oxadiazon	1.30	4.30	4.30–2000	0.995	7.1	355	71	
		Diniconazole	0.96	3.17	3.17–1000	0.995	8.5	255	51	
DµSPE-DLLME-GC-FID	Herbal infusions	Malathione	1.1	3.7	3.7–1000	0.993	1.8	455	91	21
		Haloxyfop-r-methyl	0.38	1.3	1.3–1000	0.995	2.6	305	61	
		Hexaconazole	1.5	5.1	5.1–1000	0.996	4.9	275	55	
		Oxadiazon	0.34	1.1	1.1–1000	0.995	3.4	285	57	
		Diniconazole	1.3	4.3	4.3–1000	0.996	1.0	230	46	
		Clodinafop–propargyl	0.66	2.2	2.2–1000	0.998	2.1	425	85	
		Tebuconazole	1.5	5.1	5.1–1000	0.998	3.6	330	66	
		Triticonazole	0.35	1.2	1.2–1000	0.998	2.0	195	39	
		Difenoconazole	3.0	9.9	9.9–1000	0.995	1.0	475	93	
DµSPE-DLLME-GC-FID	Vegetables	Haloxyfop-r-methyl	0.32	1.05	1.05–10 000	0.9986	1.2	445	89	22
		Hexaconazole	1.14	3.76	3.76–10 000	0.9989	3.8	275	55	
		Oxadiazon	0.31	1.02	1.02–10 000	0.9995	2.6	460	92	
		Diniconazole	1.10	3.63	3.63–10 000	0.9996	1.5	355	71	
		Clodinafop–propargyl	0.57	1.88	1.88–10 000	0.9986	2.8	400	80	
		Tebuconazole	1.21	3.96	3.96–10 000	0.9992	3.2	225	45	
		Difenoconazole	2.65	8.75	8.75–10 000	0.9989	1.1	480	96	
DµSPE-DLLME-GC-FID	Grape juice	Haloxyfop-r-methyl	2.45	8.08	8.08–6000	0.9985	0.84	495	99	This study
		Oxadiazon	2.31	7.62	7.62–6000	0.9978	2.37	465	93	
		Diniconazole	2.00	6.60	6.60–6000	0.9988	1.03	365	73	
		Tebuconazole	2.22	7.32	7.32–6000	0.9971	1.28	390	78	
		Difenoconazole	2.58	8.51	8.51–6000	0.999	1.26	480	96	

a Limit of detection (µg L^−1^).

b Limit of quantification (µg L^−1^).

c Linear Range (µg L^−1^).

d Coefficient of determination.

e Relative standard deviation (%).

f Enrichment factor.

g Extraction recovery (%).

h Headspace single drop microextraction-gas chromatography-flame ionization detection.

i Micro solid phase extraction-high performance liquid chromatography-ultraviolet detection.

j Dispersive magnetic solid phase extraction-high performance liquid chromatography-diode array detector.

k Solid phase extraction-high performance liquid chromatography-tandem mass spectrometry.

l Dispersive solid phase extraction–liquid chromatography-tandem mass spectrometry.

m Dispersive micro solid phase extraction-dispersive liquid–liquid microextraction-gas chromatography-fame ionization detection.

## 4. Conclusions

In the present study, a reliable and efficient analytical strategy based on DµSPE-DLLME coupled with GC-FID was successfully developed for the extraction and determination of five pesticide residues in grape juice samples. An organic polymer synthesized *via* a simple one-pot polyamidation reaction was employed as the sorbent, providing strong interactions with the target analytes. The synthesized material was comprehensively characterized using FTIR, XRD, SEM, BET and EDX analyses, confirming formation of the sorbent structure with suitable surface morphology and adsorption capability. Under optimized conditions, the proposed method exhibited low LODs (2.00–2.58 µg L^−1^), wide LRs, and excellent linearity (*r*^2^ ≥ 0.9971). High EFs (365–495) and ERs (73–99%) were achieved with satisfactory precision (RSD ≤ 4.42%). Matrix effect studies demonstrated negligible interference from grape juice constituents, with RRs ranging from 88 to 119%. Overall, the proposed DµSPE-DLLME-GC-FID method combined high sensitivity, excellent precision, wide LR, and strong enrichment capability with relatively low solvent consumption and instrumental simplicity. However, it should be noted that the use of a chlorinated extraction solvent limits the overall environmental friendliness of the method.

## Conflicts of interest

The authors have no relevant financial or non-financial interests to disclose.

## Abbreviation

DµSPE: Dispersive micro solid phase extraction
DLLME: Dispersive liquid–liquid microextraction
GC: Gas chromatography
FID: Flame ionization detector
EF: Enrichment factor
RSD: Relative standard deviation
LR: Linear range
LOD: Limit of detection
LOQ: Limit of quantification

## Supplementary Material

RA-OLF-D6RA01736C-s001

## Data Availability

All data generated or analyzed during this study are included in this published article and supplementary information (SI). Several results are provided within the SI. Supplementary information is available. See DOI: https://doi.org/10.1039/d6ra01736c.
