# Supplementary material for: Extraction of some pesticides from grape juice samples using an amide-based polymer as a sorbent followed by gas chromatographic determination
Source: RSC Adv. 2026 Oct 26. Online ahead of print. doi: 10.1039/d6ra01736c (PMC13702064; doi:10.1039/d6ra01736c)
Supplement: Supplementary file 1 [file RA-OLF-D6RA01736C-s001.pdf]

Supplementary Information (SI) for RSC Advances.
This journal is © The Royal Society of Chemistry

Supplementary material

Extraction of some pesticides in grape juice samples using an amide-based polymer as a sorbent followed by gas chromatographic determination

Fatemeh Ershad [a], Mir Ali Farajzadeh [a, b*], Sina Mohammad Mehri [a], Mohammad Reza Afshar Mogaddam [c, d, e]

[a] Department of Analytical Chemistry, Faculty of Chemistry, University of Tabriz, Tabriz, Iran.

[b] Engineering Faculty, Near East University, 99138 Nicosia, North Cyprus, Mersin 10, Turkey.

[c] Food and Drug Safety Research Center, Pharmaceutical Sciences Institute, Tabriz University of Medical Sciences, Tabriz, Iran.

[d] New Material and Green Chemistry Research Center, Khazar University, 41 Mehseti Street, Baku AZ1096, Azerbaijan

[e] Pharmaceutical Analysis Research Center, Pharmaceutical Sciences Institute, Tabriz University of Medical Sciences, Tabriz, Iran

*Corresponding author: M.A. Farajzadeh

Tel.: +98 41 33393084

Fax: +98 41 33340191

Email address: mafarajzadeh@yahoo.com; mafarajzadeh@tabrizu.ac.ir

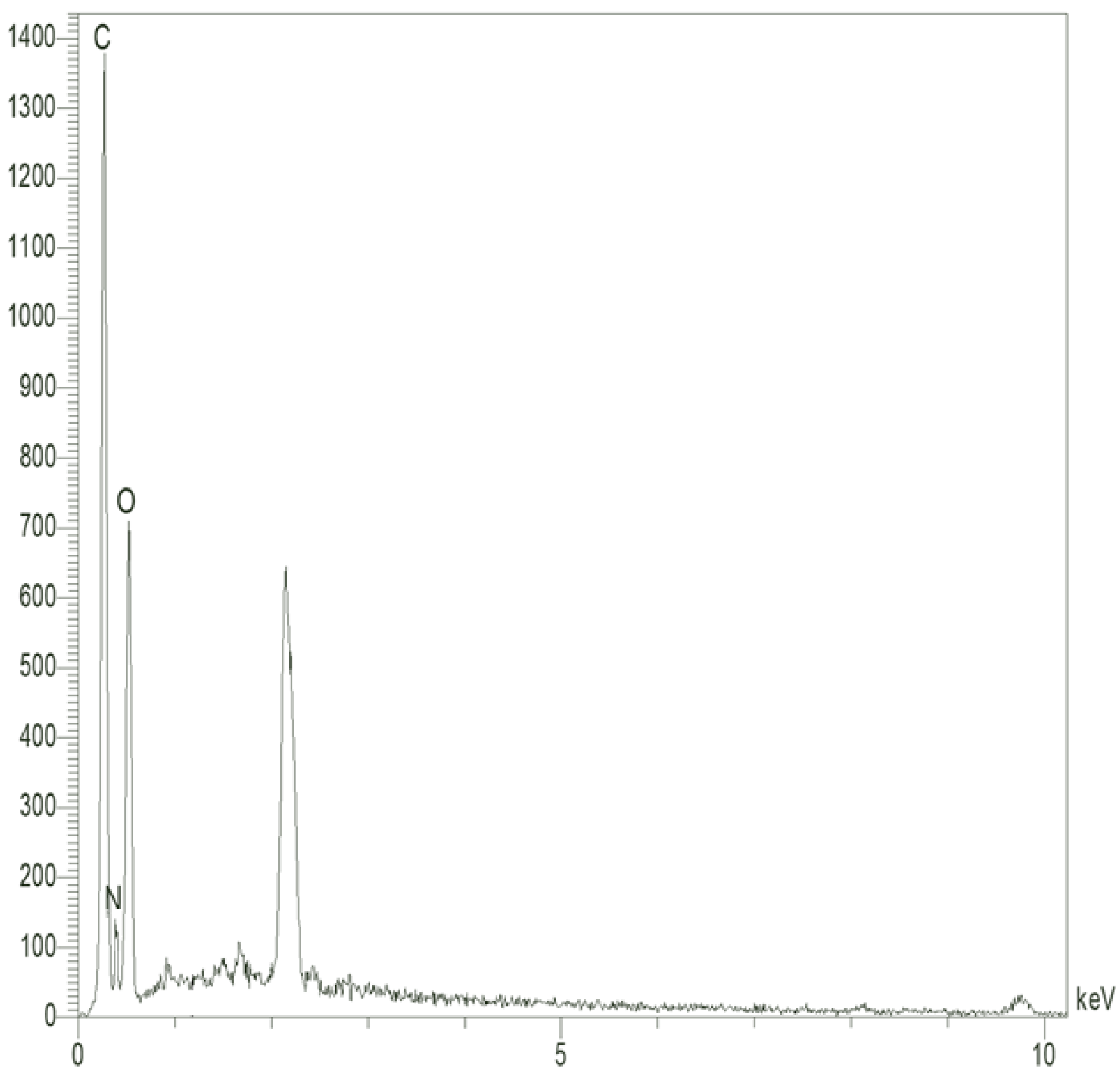

Fig. S1. EDX data of the adsorbent.

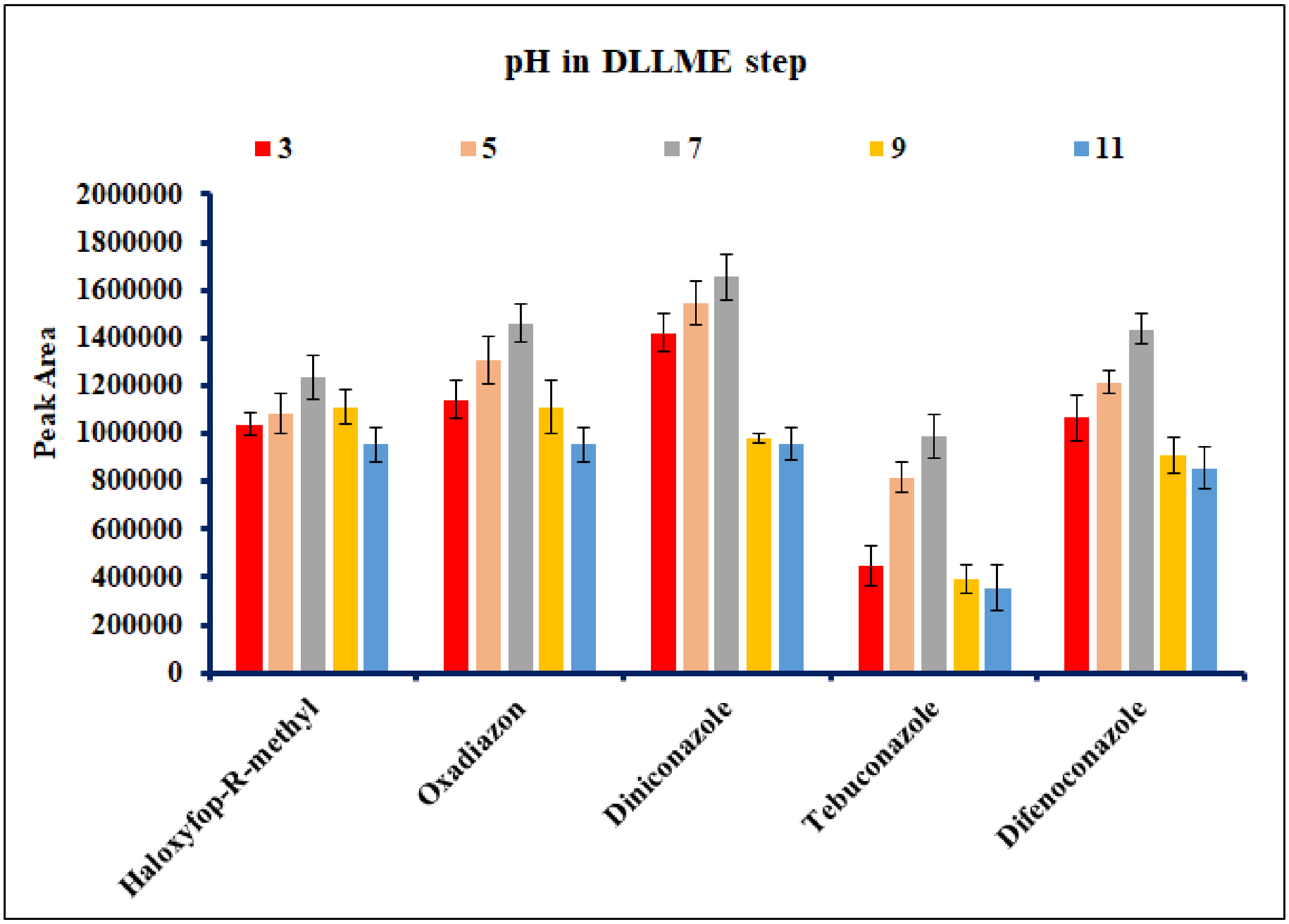

Fig. S2. Optimization of aqueous solution pH in DLLME step.

Extraction conditions were the same as those shown in Fig. 5, except that 29 μL of 1,1,1-TCE was used as the extraction solvent. Error bars depict the minimum and maximum values of three repeated determinations.

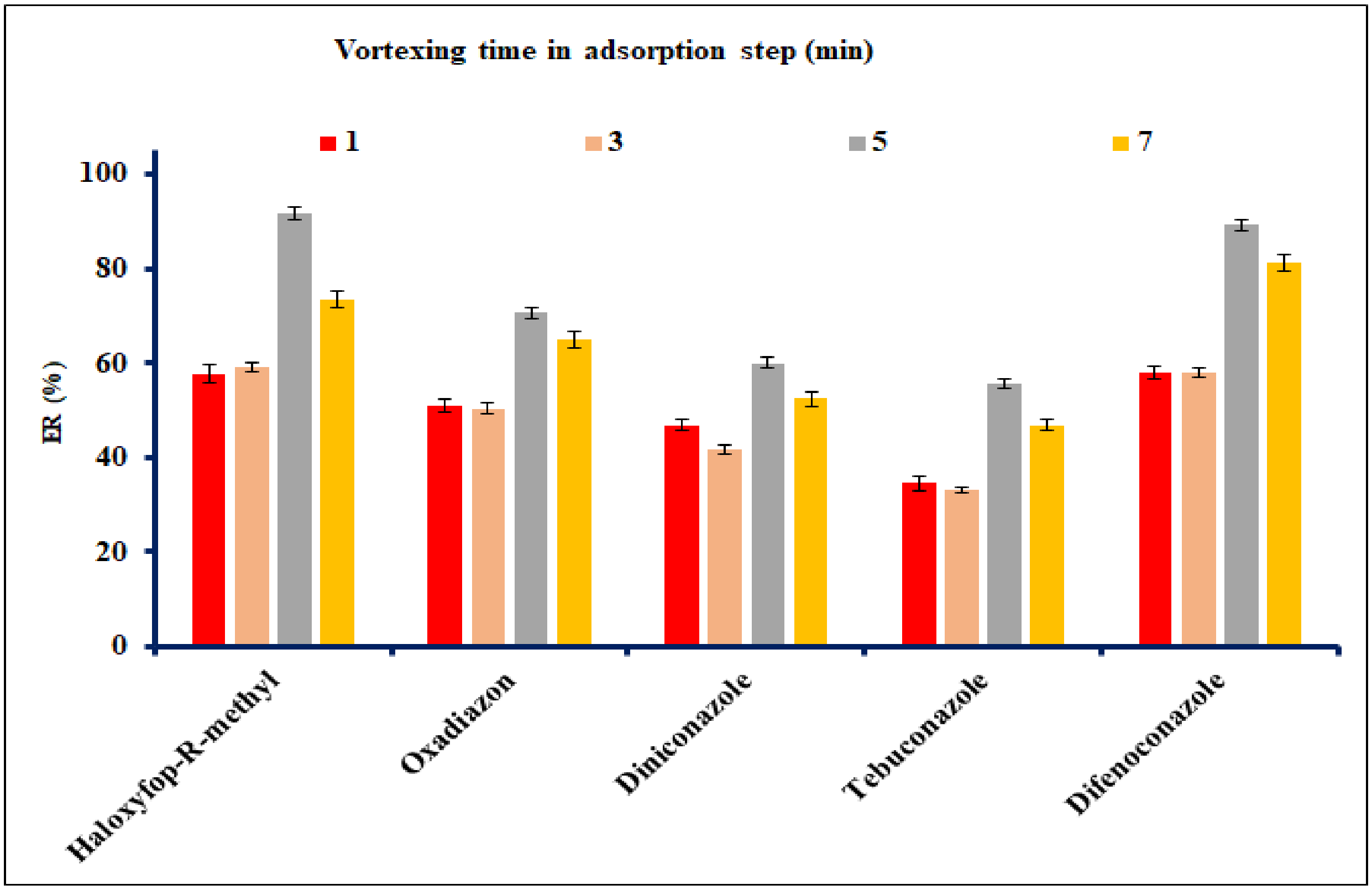

Fig. S3. Optimization of vortexing time in adsorption step.

Extraction conditions were the same as those shown in Fig. 7, except that 25% (*w/v*) Na_2SO_4 was used.

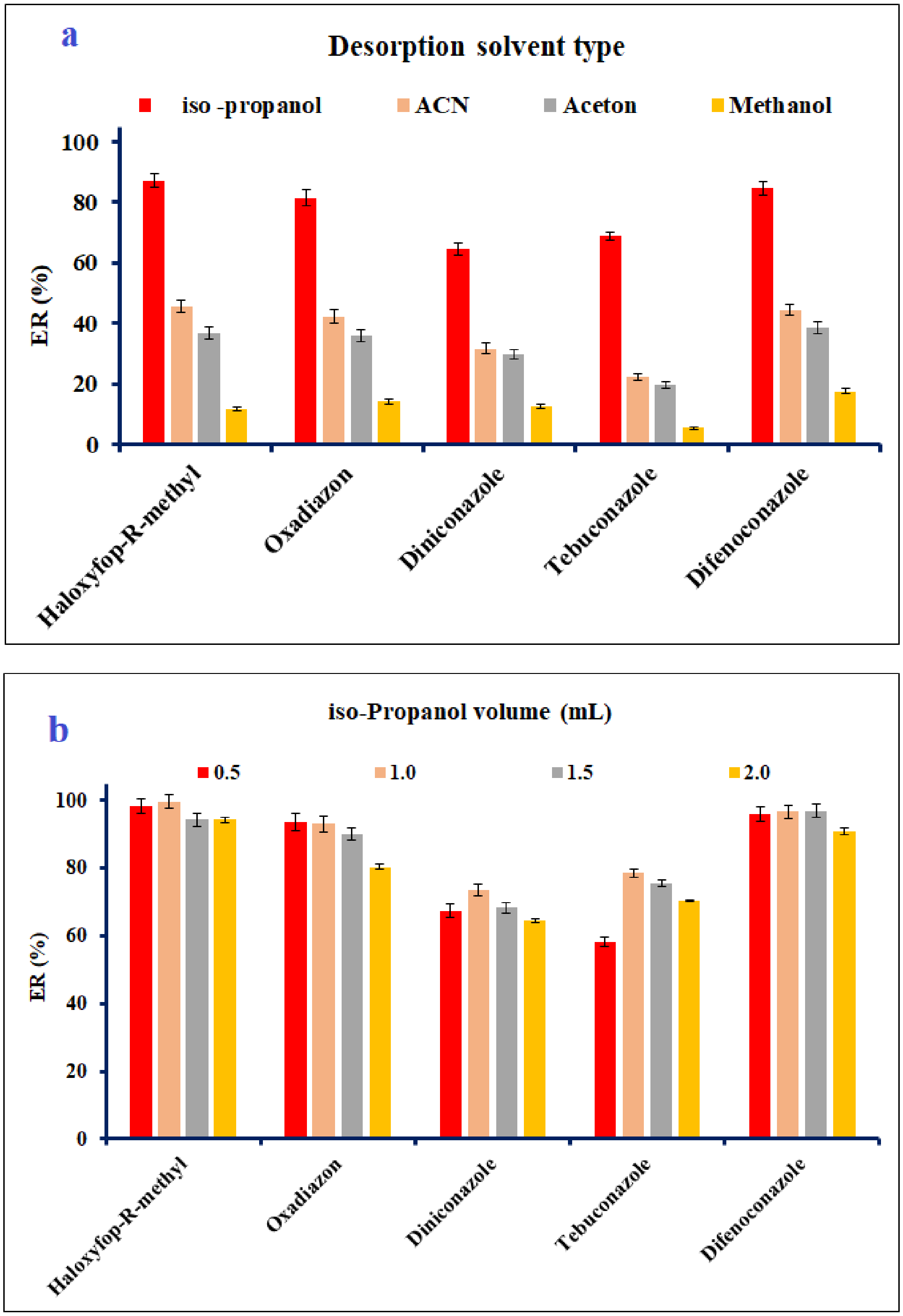

Fig. S4. Selection of desorption solvent type (a) and volume (b) in DμSPE step.

Extraction conditions were the same as those shown in Fig. S3, except that 5 min was set for vortexing time.

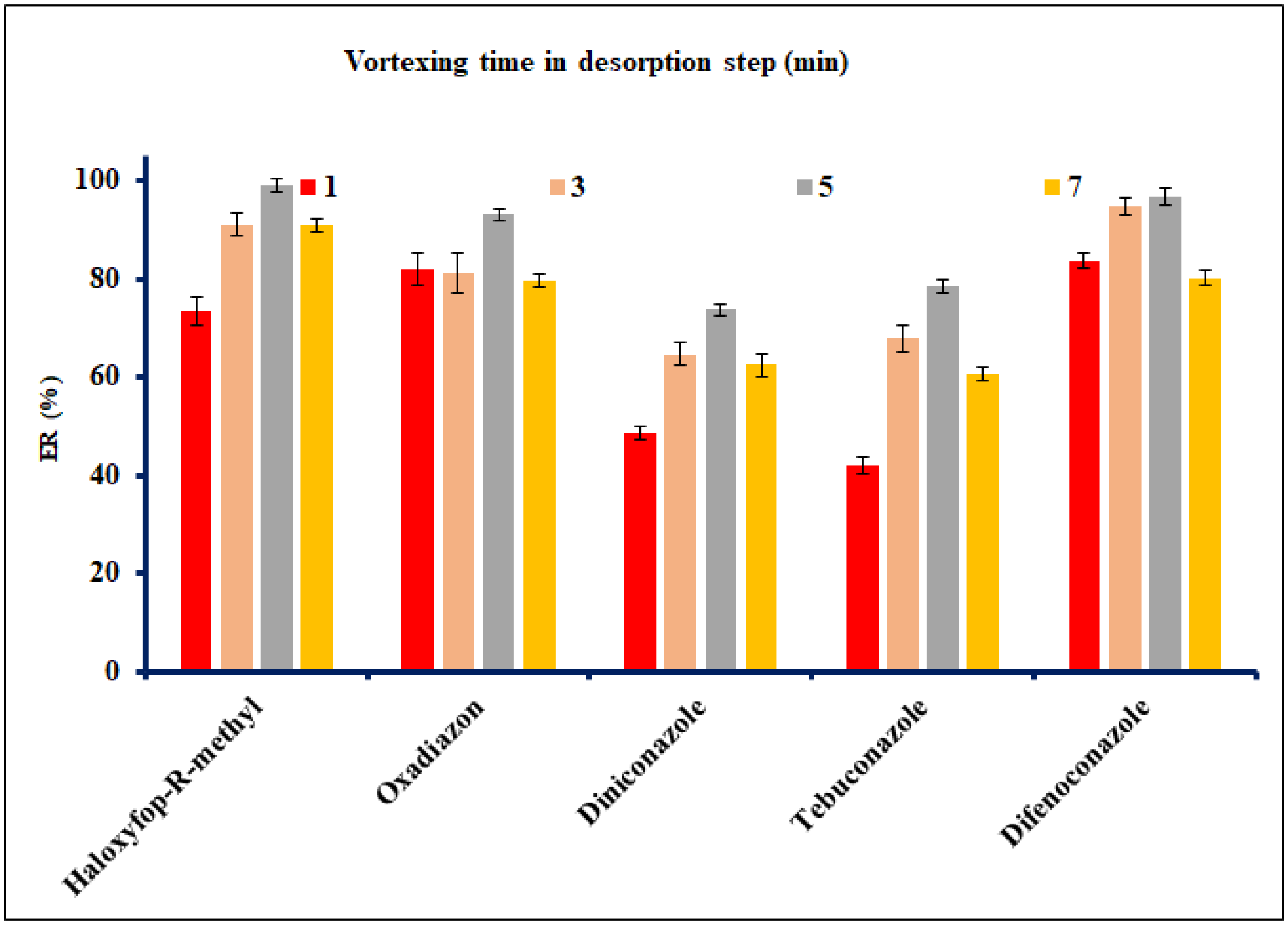

Fig. S5. Optimization of vortexing time in the desorption step.

Extraction conditions were the same as those shown in Fig. S4, except that 1.0 mL of *iso*-propanol was used as desorption solvent.

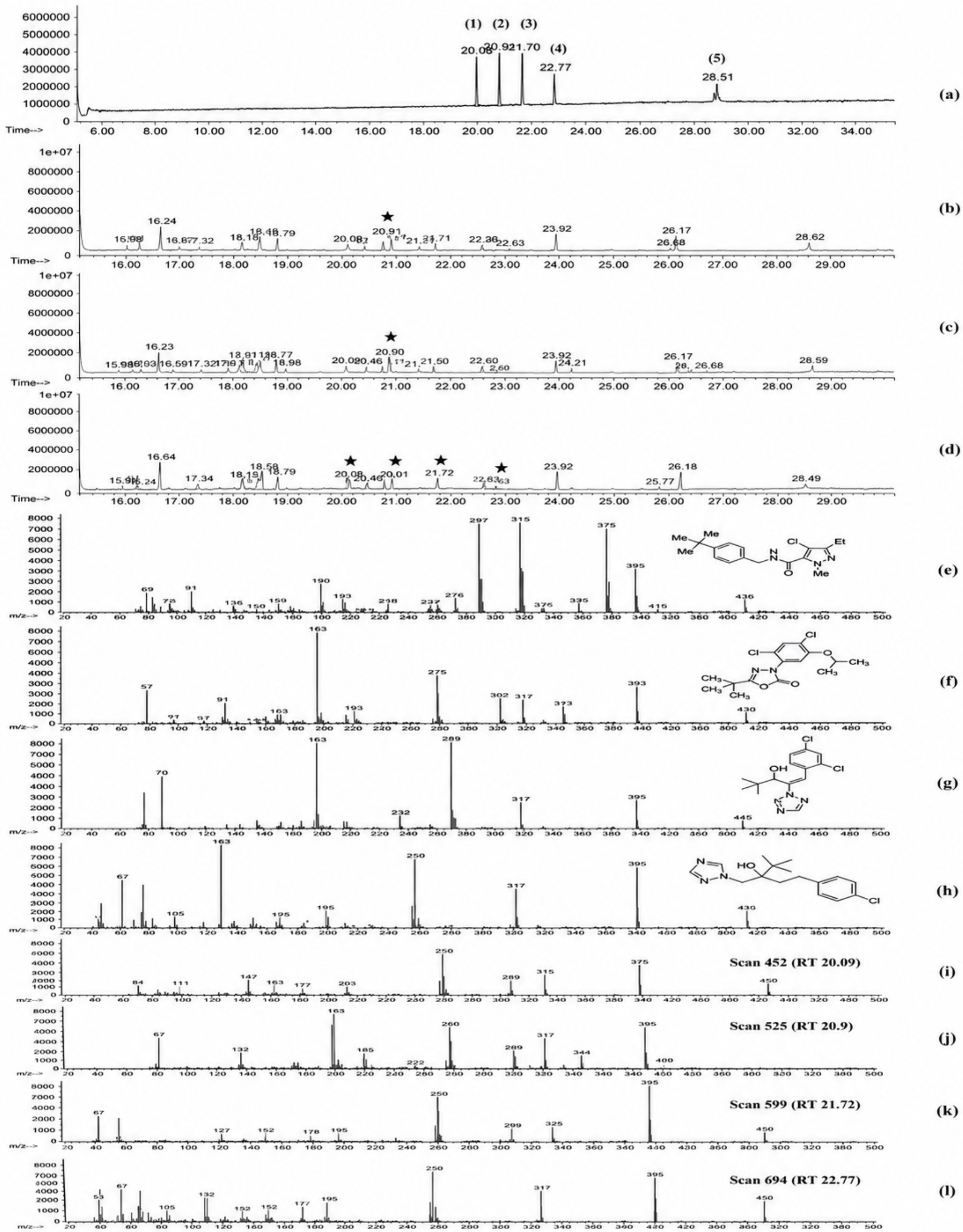
(1)
(2)
(3)
(4)
(5)
20.08
20.92
21.70
22.77
28.51
(a)
(b)
(c)
(d)
(e)
(f)
(g)
(h)
Scan 452 (RT 20.09)
(i)
Scan 525 (RT 20.9)
(j)
Scan 599 (RT 21.72)
(k)
Scan 694 (RT 22.77)
(l)
Time-->
m/z-->

Fig. S6. Total ions current chromatograms of standard solution (200 mg L^{-1} of each pesticide in methanol) (a), grape juice #1 (b), grape juice #4 (c), grape juice #2 (d), standard mass spectra of haloxyfop-R-methyl (e), oxadiazon (f), diniconazole (g), tebuconazole (h), and mass data of the compounds eluted in the retention times of analytes (i, j, k and l). Peaks identification in chromatogram (a): (1) haloxyfop-R-methyl, (2) oxadiazon, (3) diniconazole, (4) tebuconazole, and (5) difenoconazole.
